# Examining Pathways of Iron and Sulfur Acquisition, Trafficking, Deployment, and Storage in Mineral-Grown Methanogen Cells

**DOI:** 10.1128/JB.00146-21

**Published:** 2021-09-08

**Authors:** Devon Payne, Eric M. Shepard, Rachel L. Spietz, Katherine Steward, Sue Brumfield, Mark Young, Brian Bothner, William E. Broderick, Joan B. Broderick, Eric S. Boyd

**Affiliations:** a Department of Microbiology and Immunology, Montana State University, Bozeman, Montana, USA; b Department of Chemistry and Biochemistry, Montana State University, Bozeman, Montana, USA; c Department of Plant Sciences, Montana State University, Bozeman, Montana, USA; NCBI, NLM, National Institutes of Health

**Keywords:** methanogen, iron-sulfur cluster, proteomics, FeS_2_, pyrite, mackinawite, FeS, DtxR, EPR, Feo

## Abstract

Methanogens have a high demand for iron (Fe) and sulfur (S); however, little is known of how they acquire, deploy, and store these elements and how this, in turn, affects their physiology. Methanogens were recently shown to reduce pyrite (FeS_2_), generating aqueous iron sulfide (FeS_aq_) clusters that are likely assimilated as a source of Fe and S. Here, we compared the phenotypes of Methanococcus voltae grown with FeS_2_ or ferrous iron [Fe(II)] and sulfide (HS^−^). FeS_2_-grown cells are 33% smaller yet have 193% more Fe than Fe(II)/HS^−^-grown cells. Whole-cell electron paramagnetic resonance revealed similar distributions of paramagnetic Fe, although FeS_2_-grown cells showed a broad spectral feature attributed to intracellular thioferrate-like nanoparticles. Differential proteomic analyses showed similar expression of core methanogenesis enzymes, indicating that Fe and S source does not substantively alter the energy metabolism of cells. However, a homolog of the Fe(II) transporter FeoB and its putative transcriptional regulator DtxR were up-expressed in FeS_2_-grown cells, suggesting that cells sense Fe(II) limitation. Two homologs of IssA, a protein putatively involved in coordinating thioferrate nanoparticles, were also up-expressed in FeS_2_-grown cells. We interpret these data to indicate that, in FeS_2_-grown cells, DtxR cannot sense Fe(II) and therefore cannot downregulate FeoB. We suggest this is due to the transport of Fe(II) complexed with sulfide (FeS_aq_), leading to excess Fe that is sequestered by IssA as a thioferrate-like species. This model provides a framework for the design of targeted experiments aimed at further characterizing Fe acquisition and homeostasis in M. voltae and other methanogens.

**IMPORTANCE** FeS_2_ is the most abundant sulfide mineral in the Earth’s crust and is common in environments inhabited by methanogenic archaea. FeS_2_ can be reduced by methanogens, yielding aqueous FeS_aq_ clusters that are thought to be a source of Fe and S. Here, we show that growth of Methanococcus voltae on FeS_2_ results in smaller cell size and higher Fe content per cell, with Fe likely stored intracellularly as thioferrate-like nanoparticles. Fe(II) transporters and storage proteins were upregulated in FeS_2_-grown cells. These responses are interpreted to result from cells incorrectly sensing Fe(II) limitation due to assimilation of Fe(II) as FeS_aq_. These findings have implications for our understanding of how Fe/S availability influences methanogen physiology and the biogeochemical cycling of these elements.

## INTRODUCTION

Methanogens, organisms that generate methane (CH_4_) from carbon dioxide (CO_2_) and hydrogen (H_2_) and/or small organic molecules such as acetate, formate, and methanol, play key roles in the biogeochemical cycling of elements (1). Methanogens are also models for numerous biofuel applications, including as feedstocks of CH_4_ and other compounds (e.g., H_2_) that could be used as alternatives to fossil crude oil and natural gas as energy storage molecules (2). Much of the impact of methanogens on biogeochemical cycles and their utility as production platforms for energy storage molecules is attributable to the abundance and diversity of metalloproteins that support their cellular energy metabolism (1). The functioning of these metalloproteins is often dependent on both simple and complex iron-sulfur ([Fe-S]) clusters that are involved in electron transfer as well as substrate binding, activation, and catalysis (3, 4). This includes metalloproteins involved in the interconversion of H_2_ ([NiFe] hydrogenase), reduction of dinitrogen to ammonia (nitrogenase), and interconversion of carbon monoxide and CO_2_ (carbon monoxide dehydrogenase) (1, 5–7).

The dependence of methanogen metabolism on simple and complex [Fe-S] clusters is underscored by several recent studies that showed that methanogens have a large demand for Fe and S relative to other cell types (8–10). For example, Methanococcus maripaludis cells contained ∼15-fold more [Fe-S] clusters per mg protein than Escherichia coli cells (9), and the inferred proteomes of several model methanogens code for a higher number of [Fe-S] cluster binding motifs than those of facultative and obligate aerobes (10). Further, a recent analysis of >300 archaeal methanogen genomes found that roughly 5 to 8% of the total encoded proteins are predicted to bind [Fe-S] clusters (8). The greater reliance on [Fe-S] clusters in methanogen cells relative to other facultatively anaerobic or aerobic cells has been suggested to result from increased bioavailability of Fe and S in the anoxic early Earth environments (11), when the primary diversification of this group of organisms is thought to have taken place, >3.51 giga-annums (Ga) ago (12).

Contemporary methanogens typically inhabit euxinic environments that are rich in sulfide (HS^−^) and reduced metals, including Fe(II) (1). Sulfide readily reacts with metals such as Fe(II) (equation 1) and other trace metals [e.g., Ni(II), Co(II), and Zn(II)] to form precipitates with limited solubility (13).

(1) $${\text{HS}}^{-}{}_{\text{aq}}+\text{ Fe}{\left(\text{II}\right)}_{\text{aq}}\to {\text{ FeS}}_{\text{aq}}+{\text{ H}}^{+}{}_{\text{aq}}$$

In the case of Fe(II), reaction with HS^−^ initially yields soluble iron monosulfide clusters (FeS_aq_) (equation 1) (14) that can nucleate to form soluble nanoparticles with stoichiometries of up to Fe_150_S_150(aq)_ (equation 2).

(2) $${\text{Fe}}_{\text{2}}{\text{S}}_{\text{2}(\text{aq})}+{\text{ Fe}}_{\text{2}}{\text{S}}_{\text{2}(\text{aq})}\to {\text{ Fe}}_{\text{4}}{\text{S}}_{\text{4}(\text{aq})}$$

FeS_aq_ nanoparticles containing >150 FeS units precipitate as iron sulfide (FeS) solid phases such as mackinawite (FeS_mack_) (equation 3) (15).

(3) $${\text{Fe}}_{\text{15}0}{\text{S}}_{\text{15}0(\text{aq})}\to {\text{ FeS}}_{\text{mack}(\text{s})}$$

Oxidation of FeS_mack_ by sulfur compounds of intermediate oxidation state, such as polysulfide or elemental sulfur (S^0^), yields pyrite (FeS_2_) (equation 4) (16–19).

(4) $${\text{FeS}}_{\text{mack}(\text{s})}+{{\text{S}}^{0}}_{\left(\text{s}\right)}\to {\text{ FeS}}_{\text{2}(\text{s})}$$

It has also been suggested that FeS_mack_ can be oxidized by H_2_S to form FeS_2_ (equation 5) (20, 21).

(5) $${\text{FeS}}_{\text{mack},\text{s}}+{\text{ H}}_{\text{2}}{\text{S}}_{\left(\text{aq}\right)}\to {\text{ FeS}}_{\text{2}(\text{s})}+{\text{ H}}_{\text{2}(\text{g})}$$

FeS_aq_, FeS_mack_, and FeS_2_ are common in anoxic, sulfidic environments inhabited by methanogens (reviewed in references 15 and 21), and as previously mentioned, their formation proceeds via soluble (hydrated) molecular FeS_aq_ cluster intermediates (e.g., Fe_2_S_2_ · 4H_2_O) that are small (<2 nm; <150 FeS units) (18). Thermodynamic modeling indicates that the dominant form of FeS_aq_ at circumneutral to slightly alkaline pH is the uncharged FeS^0^_aq_ species, while protonated FeHS^+^_aq_ species predominate at slightly acidic pH (<6.5) (15, 22). This opens the possibility that small, dissolved, and uncharged FeS^0^_aq_ molecular clusters could theoretically diffuse across the cell membrane or, if present as charged clusters (FeHS^+^_aq_), be actively transported into cells at physiological pH. Alternatively, protein- or ligand-bound FeS_aq_ clusters could be transported into cells. Once inside the cells, FeS species could be trafficked via unknown mechanisms to meet the Fe and/or S demands associated with biosynthesis of amino acids, vitamins, [Fe-S] clusters, and other cofactors (e.g., siroheme) or coenzymes (e.g., coenzyme M [CoM]).

Recent data show that Methanococcus voltae A3 and Methanosarcina barkeri MS, representatives of early diverging hydrogenotrophic methanogens that lack cytochromes and more recently diverging methanogens that possess cytochromes (23–25), respectively, can reductively dissolve FeS_2_ and use released Fe and S to meet biosynthetic demands (26). Interestingly, during reduction of FeS_2_, HS^−^ was found to be released into the aqueous phase; however, Fe(II) was not detectable in solution. Further, addition of 1 mM HS^−^ to cultures grown with FeS_2_ had no impact on growth kinetics, and instead, cells continued to produce HS^−^ through reduction of FeS_2_. This indicates that HS^−^ is unlikely to be the primary source of S that is assimilated in cells that are reducing FeS_2_.

Previous abiotic studies conducted at high temperature (>90°C) and high H_2_ partial pressure (>8 × 10^5^ Pa) have shown that reductive dissolution of FeS_2_ (reverse of equation 4) occurs through a coupled dissolution/precipitation process, with an FeS phase most likely to be pyrrhotite (Fe_1−_*_x_*S_pyr_) forming on the surface of FeS_2_ (27). FeS_mack_ and Fe_1−_*_x_*S_pyr_ are slightly soluble (solubility product = 10^−3.5^, contingent on the availability of HS^−^ [15]), and it is possible that surface-associated FeS (either FeS_mack_ or Fe_1−_*_x_*S_pyr_) dissolves and establishes an equilibrium with the aqueous phase as FeS_aq_, which could be the species cells use to simultaneously meet Fe and S demands. In particular, in the presence of >1 μM HS^−^, as is the case in cultures of M. voltae or *M. barkeri* grown with FeS_2_ as the sole Fe and S source, the predominant form of Fe(II) in equilibrium with FeS_mack_ approaches 1 μM as FeS_aq_ species (15). The concentration of HS^−^ in the medium of M. voltae and *M. barkeri* cultures growing with FeS_2_ ranges from 5 to 30 μM (26), well above the 1 μM threshold level needed to stabilize the predominant form of Fe(II) in solution as FeS_aq_. Previous studies have also suggested the possibility that FeS_aq_ is the form of Fe and S assimilated by methanogens (28). Nonetheless, it is not clear what the consequences of potential assimilation of Fe and S (as FeS_aq_) at an ∼1:1 stoichiometric ratio may be, given that cells need more S than they do Fe (9). Further, it is unknown whether cells acclimate to use less Fe or S when grown with mineral sources of Fe/S compared to growth with Fe(II)/HS^−^ and whether this, in turn, alters the distribution of Fe and S in cells. Addressing such questions has consequences for understanding the sources and sinks of Fe and S in euxinic environments inhabited by methanogens and could provide new insights into potential limitations imposed by Fe and S availability and speciation on the activity and function of such cells in natural systems.

In the present study, we examined M. voltae A3 cells grown with formate in minimal base salts medium provided with either Fe(II)/HS^−^ or FeS_2_ as the sole source of Fe and S. Electron microscopy was used to identify morphological differences between cells. Cells were analyzed for differences in cellular Fe content using atomic absorption (AA) spectroscopy and were examined for variation in the abundance and composition of [Fe-S] clusters using electron paramagnetic resonance (EPR) spectroscopy. Finally, cells were subjected to differential proteomics analyses to identify similarities and differences in the abundances of proteins involved in core methanogenesis pathways, those involved in Fe acquisition/homeostasis, and those that bind [Fe-S] clusters as indicators of potential changes in energy metabolism, Fe/S acquisition, and Fe/S deployment, respectively. Results are discussed in terms of potential pathways for assimilating FeS_aq_ and the consequences for growth with soluble versus mineral forms of Fe and S in terms of sources and sinks of Fe and S.

## RESULTS AND DISCUSSION

### Cell morphology and size.

**FIG 1:**
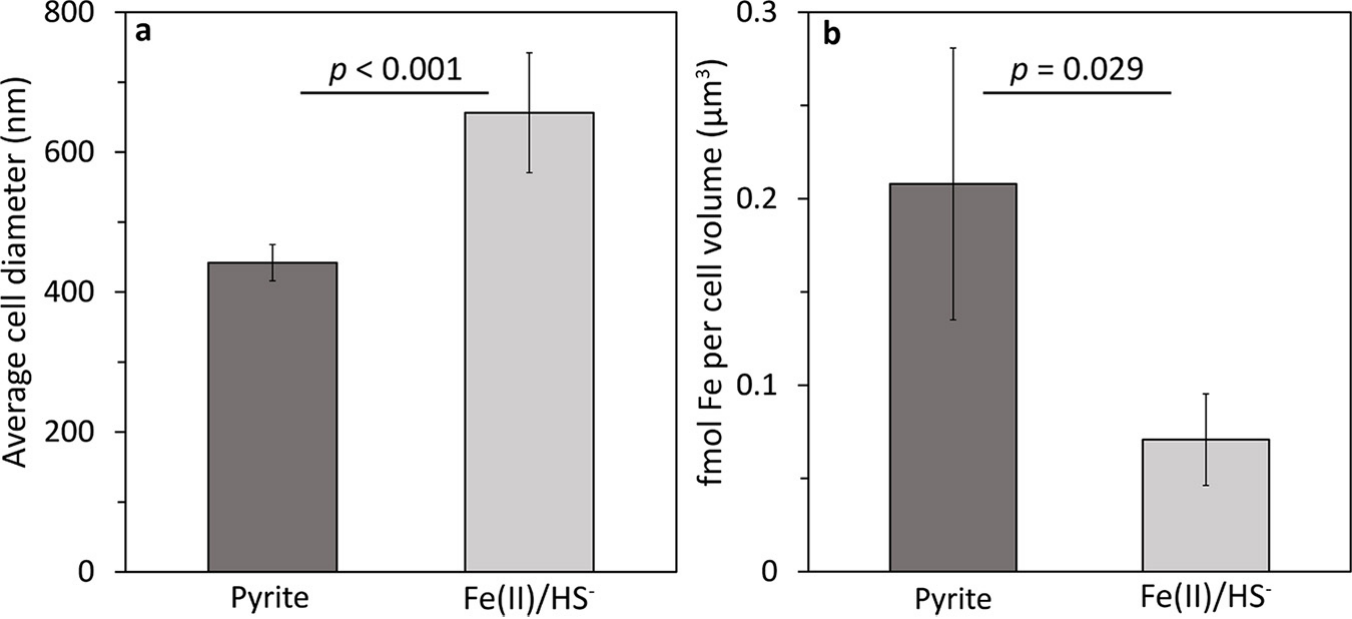
Cell size and iron (Fe) content of Methanococcus voltae grown with different Fe and sulfur (S) sources. M. voltae was grown in base salts medium with either synthetic pyrite nanoparticles or ferrous iron [Fe(II)] and sulfide (HS^−^) as the sole Fe and S sources. Cell size was determined for each condition using transmission electron microscopy (TEM) (a). Fe content per cell unit volume (assuming coccoid morphology based on TEM measurements) was determined by atomic absorption spectroscopy (b). Data in panel a are means and standard deviations of measured cell diameters from three fields of view for each condition. Data in panel b are means and standard deviations for four biological replicates. Statistical significance was calculated using Student’s two-tailed *t* test; *P* values are in the figure.

Cultures of M. voltae grown with formate as the electron donor and provided with either Fe(II)/HS^−^ or FeS_2_ as the sole Fe and S source were examined using transmission electron microscopy (TEM). While the overall shape of cells did not differ substantively between treatments (i.e., all were irregular, elongated coccoids), clear differences were observed in the size of cells, with Fe(II)/HS^−^- and FeS_2_-grown cells being roughly 675 ± 85.6 nm and 450 ± 25.9 nm in diameter, respectively **(**Fig. 1a; also, see Fig. S1 in the supplemental material). This nearly 33% decrease in diameter in FeS_2_-grown cells related to Fe(II)/HS^−^-grown cells is substantial and, in bacteria, such as Escherichia coli and Bacillus subtilis, has been suggested to reflect slower growth kinetics associated with the smaller cells (29–31). More recent studies indicate that cell size is more likely a function of nutrient availability, which is in turn inextricably linked with growth kinetics (reviewed in reference 32). In our previous work with M. voltae, we observed similar growth kinetics and yields when cells were cultured with formate as the electron donor and with Fe(II)/HS^−^ or FeS_2_ as the sole Fe and S source (26). For example, the yields of cells cultivated with Fe(II)/HS^−^ or FeS_2_ as the sole Fe and S source were 1.21 × 10^13^ and 1.39 × 10^13^ cells mol^−1^ CH_4_, respectively. We performed an experiment to convert cell number to cell dry weight in Fe(II)/HS^−^- or FeS_2_-grown cells that were separated from minerals (as described below) and found that Fe(II)/HS^−^-grown cells achieved a mass of 0.111 ± 0.005 μg (dry weight) per million cells, while FeS_2_-grown cells achieved a mass of 0.094 ± 0.010 μg (dry weight) per million cells. Despite methanogens requiring a larger amount of S than Fe (9), we noted that HS^−^ accumulated in the growth medium during growth on FeS_2_ (26). This indicated that cells were likely replete with S during growth on FeS_2_ but may be limited for another nutrient, such as Fe.

In addition to decreased size, cells that are experiencing nutrient limitation oftentimes acclimate by reducing their dependence on those limiting nutrients to assemble biomolecules (reviewed in reference 33). Perhaps the best example is the human pathogen Borrelia burgdorferi, which is responsible for Lyme disease. B. burgdorferi overcomes the host defense of limiting the availability of Fe in tissues and fluids by eliminating the use of Fe, with individual B. burgdorferi cells estimated to contain <10 Fe atoms cell^−1^, through what has been described as an Fe-sparing response (34). Methanogens apparently acclimate similarly. For example, diazotrophic organisms, including methanogens (35), can switch from using a molybdenum (Mo)-dependent nitrogenase to an alternative Mo-independent nitrogenase when Mo is limiting (36, 37). Likewise, the genome of Methanosarcina acetivorans has two different gene clusters that encode Mo and tungsten (W)-dependent forms of formylmethanofuran dehydrogenase (Mfr), possibly to allow cellular metabolism to continue when either Mo or W is limiting (38). Finally, methanogens have been shown to acclimate to nickel (Ni) limited conditions by replacing their F_420_-reducing [NiFe] hydrogenase with Ni-independent [Fe] hydrogenase and an F_420_-dependent methylenetetrahydromethanopterin dehydrogenase (5, 39, 40). These precedents prompted experiments to examine Fe usage in M. voltae cells.

### Cell iron content.

Based on the above observations, we hypothesized that M. voltae cells, when grown with FeS_2_, are Fe limited and would minimize the amount of Fe used when grown on FeS_2_. To examine this hypothesis, we compared the Fe content in M. voltae cells provided with Fe(II)/HS^−^ or FeS_2_ as their sole source of Fe and S. Prior to this analysis, we first developed and validated a technique to separate cells from minerals, so that the latter would not confound measurements of per-cell Fe content. Briefly, cells grown with Fe(II)/HS^−^ were harvested at mid-log phase and were enumerated via light microscopy. Roughly 2.3 × 10^10^ cells were then incubated anaerobically for 60 min in fresh anoxic base salts medium or fresh anoxic base salts medium containing 2 mM synthetic nanoparticulate FeS_2_. Cells were then harvested and subjected to cell separation, washing, and quantification, as detailed in Materials and Methods. Following separation, the total number of cells recovered was similar among treatments (4.12 × 10^9^ and 3.81 × 10^9^ cells for unamended and FeS_2_-amended conditions, respectively). A separate aliquot of cells was subjected to digestion with 10% nitric acid, and the lysate was subjected to an analysis of the total Fe content per cell using AA spectroscopy. The amount of Fe per cell was not significantly different between conditions (average of 8 and 11 amol Fe per cell for unamended and FeS_2_ conditions, respectively) (Fig. S2). Further, we did not detect FeS_2_ nanoparticles associated with cells during counting via light microscopy following separation, nor did we detect paramagnetic FeS_2_ signals in whole-cell EPR analyses (discussed below). Together, this indicates that our separation protocol is likely sufficient to remove residual FeS_2_, thereby mitigating the confounding effects that this would have on per-cell Fe analyses.

Following separation of four batches of Fe(II)/HS^−^- or FeS_2_-grown cells from mineral and following biomass digestion with 10% nitric acid, the amount of Fe per cell was determined via AA. While the amount of Fe was not significantly different in cultures grown with Fe(II)/HS^−^ or FeS_2_ on a per-cell basis, when normalized to per unit cell volume, cells cultivated with FeS_2_ contained significantly more (*P = *0.03) Fe than those cultivated with Fe(II)/HS^−^ (Fig. 1b; Table S1). Specifically, there was a 193% increase in the amount of Fe recovered per unit cell volume in FeS_2_-grown cells compared to those grown with Fe(II)/HS^−^. This was a surprising finding, considering that FeS_2_-grown cells are substantially smaller (which may indicate nutrient limitation) and Fe and S are in excess for both growth conditions. However, the stoichiometries of these elements are different between conditions, with the FeS_2_ condition having an Fe:S stoichiometry (1:2) that would seem to favor S limitation while the Fe(II)/HS^−^ has an Fe:S stoichiometry (1:80) that could be expected to lead to Fe limitation. These unexpected observations prompted additional analyses to identify potential differences in the trafficking, deployment, and storage of Fe in FeS_2_- versus Fe(II)/HS^−^-grown cells.

### Whole-cell EPR.

**FIG 2:**
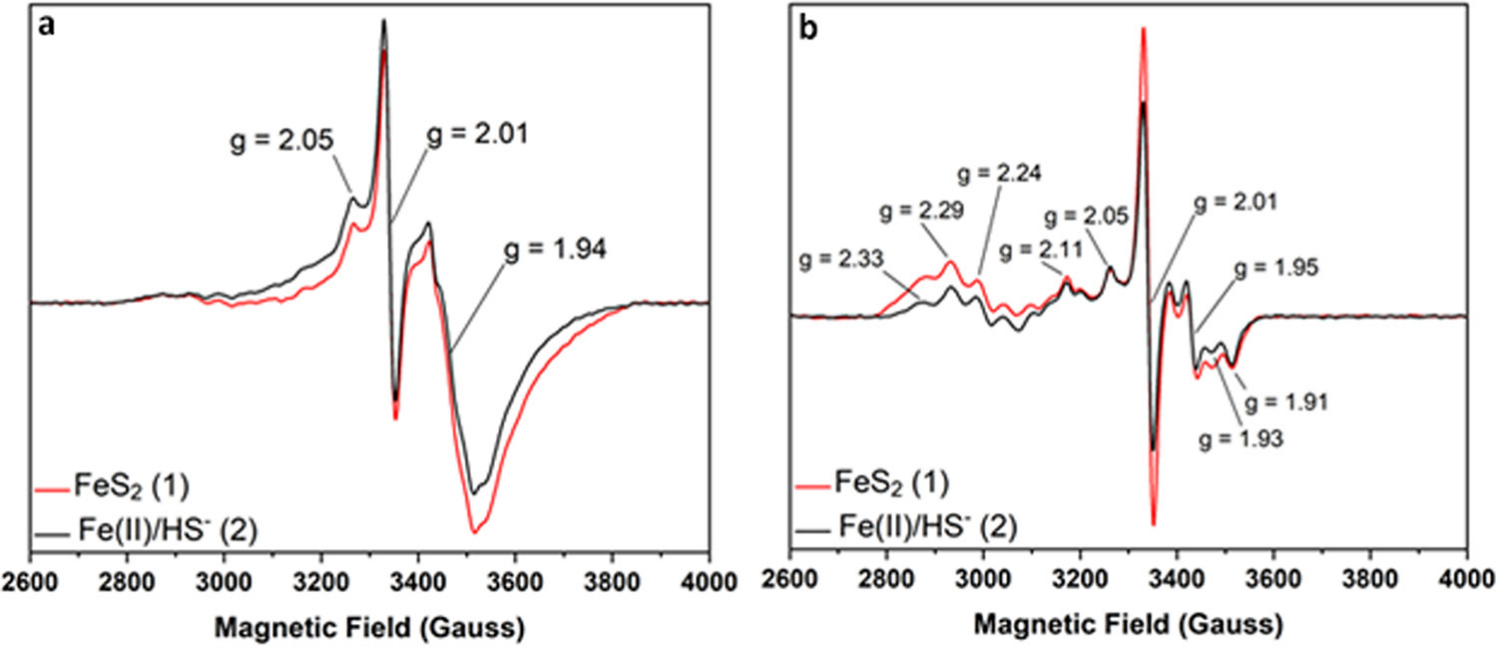
Electron paramagnetic resonance (EPR) spectra for Methanococcus voltae whole cells grown with pyrite or ferrous iron and sulfide. Cells were grown with formate and either synthetic pyrite nanoparticles (FeS_2_) or ferrous iron and sulfide [Fe(II)/HS^−^] and then separated from mineral species as described in the text. Data were collected at X-band at both 12 K (a) and 50 K (b) at 5 mW microwave power. The spectra shown in this figure have been baseline and cavity corrected in order to directly overlay the spectroscopic [Fe-S] cluster signals between the different growth conditions. Samples shown are designated in Table S1.

To provide insight into possible destinations for Fe in Fe(II)/HS^−^- versus FeS_2_-grown cells, we applied EPR spectroscopy to whole-cell samples. These samples were a larger subsample of the same cells that were treated to remove residual FeS_2_ nanoparticles, if present, and used to quantify Fe content via AA as described above (Table S1). We collected EPR spectra from the base salts medium as a control and observed limited background signals near a *g* factor value of ∼2 (Fig. S3). We also collected EPR spectra from synthetic FeS_2_ and FeS_mack_ and observed unique, broad paramagnetic signals that are absent in the whole-cell EPR spectra, suggesting that the whole-cell–mineral separation techniques were effective in removing mineral (Fig. S4). Low-temperature EPR spectral analysis of Fe(II)/HS^−^- and FeS_2_-grown M. voltae whole cells revealed highly similar, axial shaped signals with *g* values spanning the 1.94-to-2.05 region in both sample treatments (Fig. 2a). EPR spin quantitation of these signals revealed that Fe(II)/HS^−^-grown cell samples averaged 16.0 ± 1.1 μM spin (∼0.5% total Fe) and FeS_2_-grown cell samples averaged 20.3 ± 10.4 μM spin (∼1.3% total Fe). EPR spectra were collected at a range of temperatures to provide further insights into the origins of these signals. Substantial signal intensity loss was observed for the 1.94–2.05 features as the temperature was increased from 12 to 30 K, and these signals were not observed at 50 K (Fig. 2b; Fig. S2). The axial line shape, the *g* values, and the strongly temperature-dependent relaxation properties support the assignment of these features primarily to biological [4Fe-4S]^+^ cluster species (4, 41–45).

These samples additionally show evidence in the low-field region for integer spin species, perhaps a rubredoxin-like Fe(II), with *g* values of ∼10. Signals having the characteristic line shape, *g* values (*g* = ∼4 to 6), and temperature relaxation properties of *S* = 3/2 [4Fe-4S]^+^ clusters are also observed (Fig. S5) (44, 46–49). EPR spectra of Fe(II)/HS^−^- and FeS_2_-grown M. voltae whole-cell samples at higher temperatures (50 and 75 K) revealed several overlapping signals in both sample treatments, with *g* values spanning the 1.91–2.33 region (Fig. 2b). Given the *g* values and temperature dependence, these signals may arise from species such as the F_430_ nickel porphinoid of methyl CoM reductase, the noncubane [4Fe-4S] clusters in heterodisulfide reductase, and the molybdenum guanine dinucleotide cofactor of Mfr (50–55). The rich complexity of the signals likely also reflects several additional metallocofactor species, as well as biological [2Fe-2S]^+^ clusters (41, 43, 56, 57). The observation of rubredoxin-like and [4Fe-4S]^+^ cluster species in these samples is not unexpected, given the abundance of proteins with [Fe-S] motifs in methanogens (8, 10) and their detection in proteomics data from these cells (described below). Collectively, these data show that cells growing with Fe(II)/HS^−^ or FeS_2_ synthesize a similar array of proteins that have similar abundances and compositions of EPR-detectable [Fe-S] clusters.

**FIG 3:**
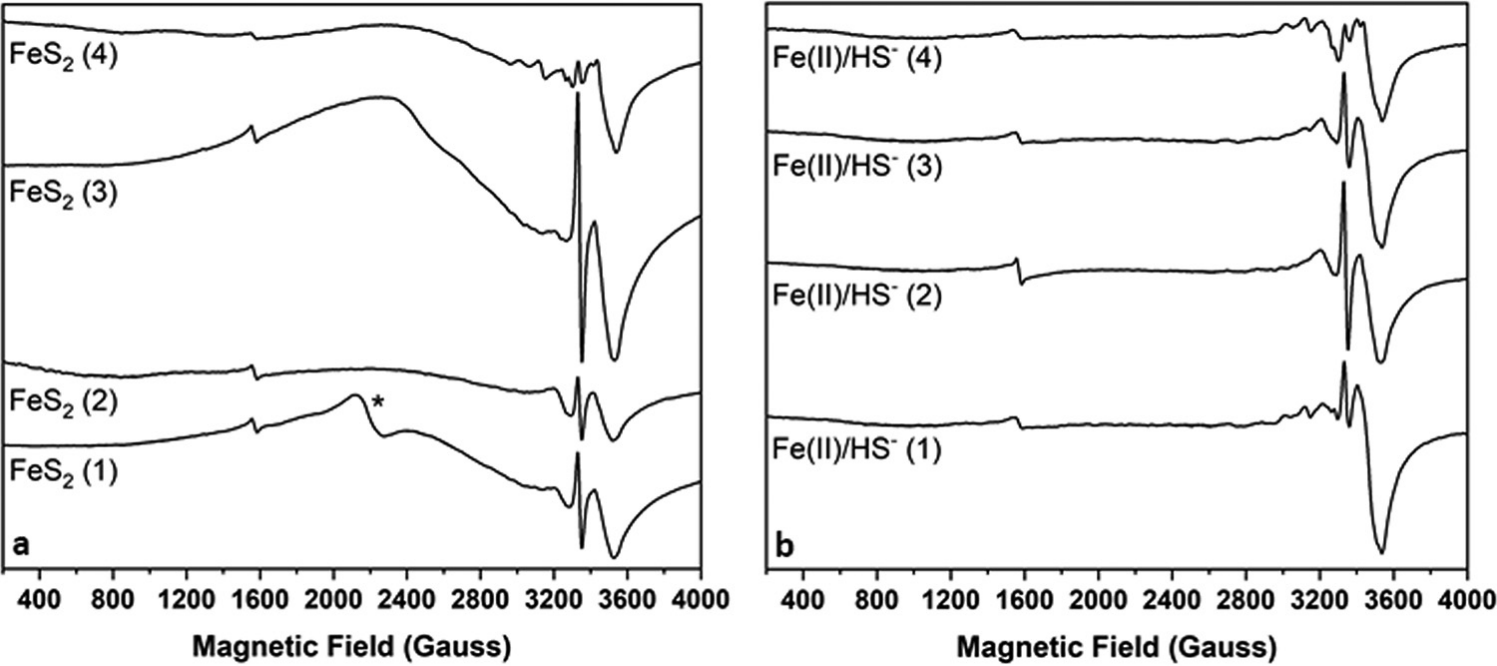
Broad-field EPR spectra for Methanococcus voltae whole-cell samples grown with either pyrite or ferrous iron and sulfide. Cells were grown with formate and either synthetic pyrite nanoparticles (FeS_2_) (a) or ferrous iron and sulfide [Fe(II)/HS^−^] (b) and then separated from mineral species as described in the text. Data were collected at X-band, with 5 mW microwave power and a temperature of 8 K. The spectra shown in this figure have not been baseline or cavity corrected. The cavity contribution to the signals near a *g* value of ∼2.0 is negligible (see the supplemental material). The asterisk denotes a paramagnetic species (*g*, ∼3.0) of unknown origin. Samples shown are designated in Table S1.

**FIG 4:**
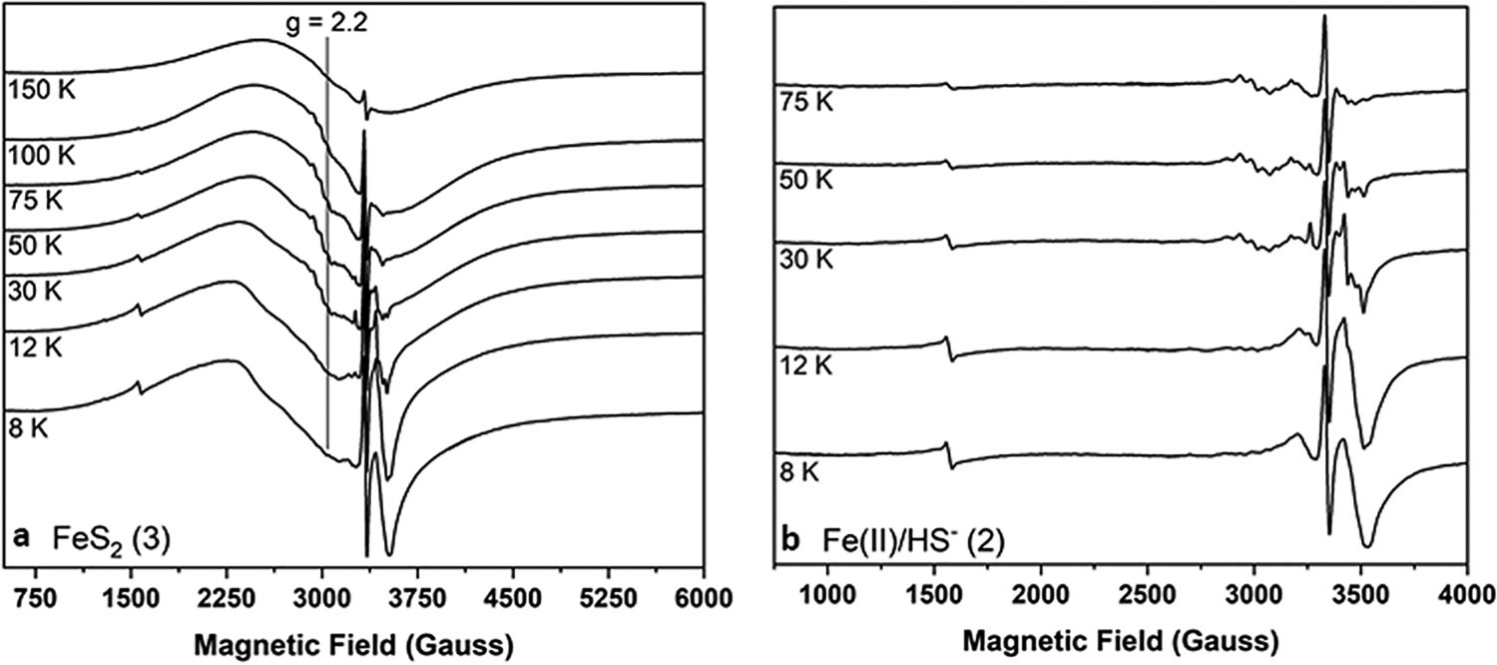
Variable-temperature, continuous-wave (CW) X-band electron paramagnetic resonance (EPR) for Methanococcus voltae whole cells grown with formate and either synthetic pyrite (FeS_2_) nanoparticles (a) or ferrous iron [Fe(II)] and sulfide (HS^−^) (b). Whole cells were characterized at 5 mW microwave power as a function of increasing temperature. Importantly, the spectra shown in this figure have not been baseline or cavity corrected. The cavity contribution to the signals near a *g* value of ∼2.0 is negligible (see the supplemental material), and we endeavored to preserve the baseline signatures in all data sets due to the variable intensity of thioferrate-like signals. Samples shown are designated in Table S1.

EPR spectra of FeS_2_-grown M. voltae whole-cell samples collected over a wide magnetic field revealed additional broad signals of variable intensity spanning ∼3,000 G and centered near a *g* value of 2.2 (Fig. 3a). This broad signal undergoes gradual intensification as the temperature is raised from 8 K to 100 K, before relaxing substantially when temperature is further increased to 150 K (Fig. 4a). The breadth of the EPR signal and its temperature dependence are largely consistent with those described for the Fe-S storage protein A (IssA) from the archaeon Pyrococcus furiosus (58). IssA stores Fe and S as linear (FeS_2_^−^)*_n_* thioferrate polymer nanostructures, and EPR spectroscopic characterization of purified IssA-thioferrate complexes reveals signal intensification up to 250 K, behavior that is consistent with synthetic potassium thioferrate species (59). The highly similar broad EPR signal observed here for FeS_2_-grown M. voltae whole cells (Fig. 4a), together with the different temperature dependence reported herein, could indicate that these cells accumulate a different type of Fe-S nanostructure, similar to but distinct from the thioferrate structures assembled in *P. furiosus* with IssA. Further, the broad signal we observe accounts for appreciable spin, estimated at between ∼100 and ∼610 μM paramagnetic species (10 to 23% total Fe) based on double integration of the EPR signal. Together with the evidence that FeS_2_-grown cells contain nearly double the Fe per unit volume relative to cells grown on Fe(II) and HS^−^, the broad EPR signal is consistent with M. voltae accumulating and storing Fe, perhaps as a type of Fe-S nanostructure. Importantly, Fe(II)/HS^−^-grown M. voltae cells exhibit either very low or undetectable amounts of this broad signal (Fig. 3b and 4b; Fig. S6), indicating that the putative thioferrate-like nanoparticles do not accumulate to nearly the same extent as they do in FeS_2_-grown M. voltae cells (Fig. 3).

### Differential proteomics analyses.

To further characterize differences in the core energy metabolism and potential protein destinations for Fe in Fe(II)/HS^−^- or FeS_2_-grown M. voltae cells, a differential proteomics approach was undertaken. We first conducted an experiment to confirm that the presence of FeS_2_ does not influence the recovery of protein following extraction, using a design similar to that used for whole-cell AA/EPR analysis. Briefly, Fe(II)/HS^−^-grown cells were harvested, and the pelleted biomass was split for incubation with sterile fresh medium with or without 2 mM FeS_2_ added. The samples were incubated on ice for 30 min, the cells were reharvested, and the resultant cell pellets were subjected to protein extraction. SDS-PAGE analysis showed no noticeable differences in protein banding patterns between the two conditions (Fig. S7), and protein quantification via NanoDrop indicated similar protein recoveries (averages of 2.95 and 2.90 mg protein ml^−1^ for cells incubated with FeS_2_ versus those not incubated with FeS_2_, respectively). These observations indicate that the presence of FeS_2_ does not substantively interfere with the composition or abundance of proteins following extraction.

**FIG 5:**
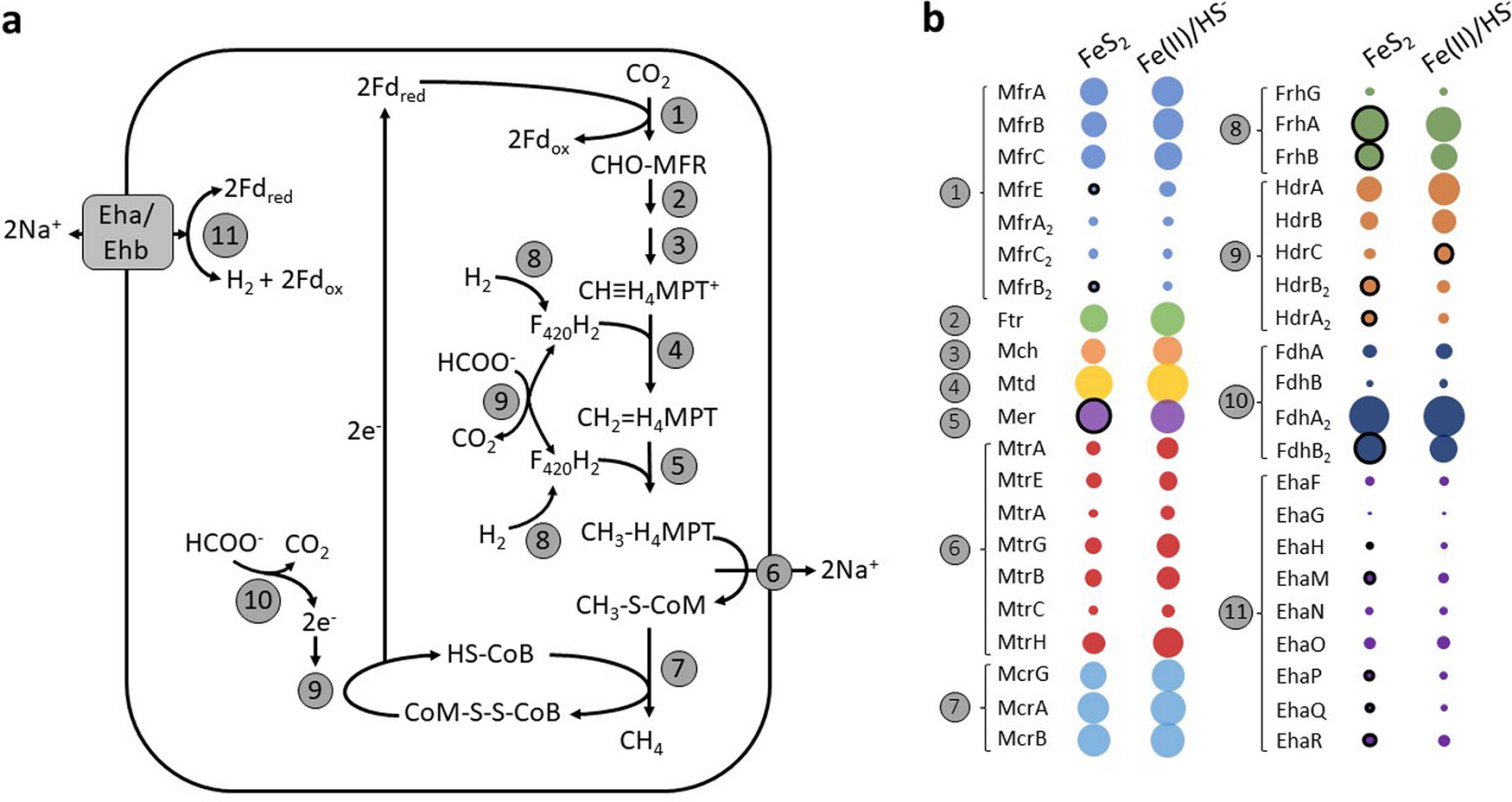
Abundances of core methanogenesis pathway proteins in Methanococcus voltae cells grown with different iron (Fe) and sulfur (S) sources. Proteins were extracted from log-phase M. voltae cells provided with formate and synthetic pyrite nanoparticles (FeS_2_) or ferrous iron [Fe(II)] and sulfide (HS^−^). A model for methanogenesis supported by formate, including replenishment of intermediates through membrane-bound and ion-translocating [NiFe] hydrogenase (Eha/Ehb), is shown in panel a. The relative abundances of key methanogenesis proteins for reactions identified in panel a are presented for FeS_2_ and Fe(II)/HS^−^ conditions in panel b. Bubble sizes for each protein in panel b represent the mean of the normalized spectral intensity of three biological replicates per condition. Bubbles are colored based on function, with black outlines indicating proteins that are significantly (*P < *0.05; log_2_ FC > 1) up-expressed under one condition relative to the other. Enzyme names and abbreviations for each step in methanogenesis are as follows: 1, formylmethanofuran dehydrogenase (Mfr); 2, formylmethanofuran:tetrahydromethanopterin (H_4_MPT) formyltransferase (Ftr); 3, methenyltetrahydromethanopterin cyclohydrolase (Mch); 4, F_420_H_2_-dependent methylenetetrahydromethanopterin dehydrogenase (Mtd); 5, coenzyme F_420_-dependent *N*^5^,*N*^10^-methylenetetrahydromethanopterin reductase (Mer); 6, methyl-H_4_MPT–coenzyme M methyltransferase (Mtr); 7, methyl coenzyme M reductase (Mcr); 8, coenzyme F_420_-reducing [NiFe] hydrogenase (Frh); 9, heterodisulfide reductase (Hdr); 10, formate dehydrogenase (Fdh); 11, energy-converting [NiFe] hydrogenase (Eha/Ehb).

M. voltae was grown with synthetic nanoparticulate FeS_2_ or Fe(II)/HS^−^, and cell CH_4_ concentrations were determined before proteins were extracted from cells in mid-log phase (Table S2). Differential proteomics analyses identified broad differences in the global proteome of M. voltae between these conditions (Fig. S8). We then narrowed our analyses to the abundance of proteins that are involved in the core pathway of methanogenesis (1, 60) to determine if growth on FeS_2_ had an influence on the overall energetic state of cells. Broadly, we did not detect substantive differences in many of the core proteins for methanogenesis between cells growing with Fe(II)/HS^−^ and those growing with FeS_2_ (Fig. 5; Data Set S1). However, heterodisulfide reductase subunits HdrB_2_ and HdrA_2_, the F_420_-reducing hydrogenase subunits FrhA and FrhB, and one subunit of a second copy of formylmethanofuran dehydrogenase, MfrB_2_, were slightly up-expressed under the FeS_2_ growth condition compared to the Fe(II)/HS^−^ growth condition. Conversely, MfrE and HdrC were slightly down-expressed in cells grown on FeS_2_ relative to those grown on Fe(II)/HS^−^. While these subtle differences represent changes in expression of several subunits, no complete enzyme complex involved in methanogenesis displayed differential expression. Thus, the energy metabolism of M. voltae appears to be similar when cells are grown with Fe(II)/HS^−^ and FeS_2_.

**FIG 6:**
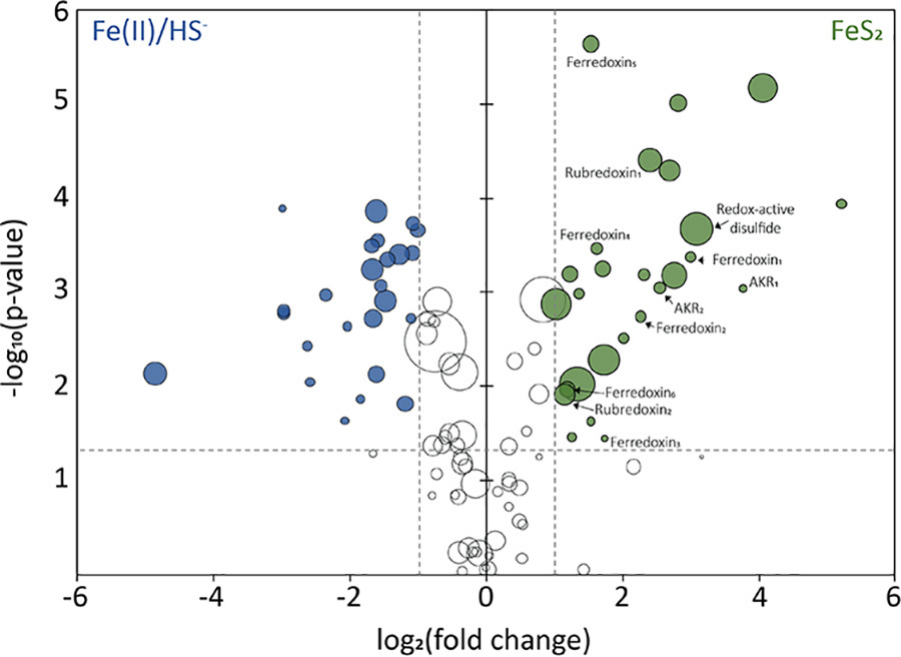
Differential expression of proteins predicted to bind iron-sulfur ([Fe-S]) clusters in Methanococcus voltae. M. voltae was grown to mid-log phase with formate and synthetic pyrite (FeS_2_) nanoparticles or ferrous iron [Fe(II)] and sulfide (HS^−^) as sole iron and sulfur sources. The data presented are limited to proteins that are predicted to bind [Fe-S] clusters, as determined with MetalPredator (61), in the proteomics data set. Along the *x* axis, a positive log_2_ FC is associated with up-expression of proteins in FeS_2_-grown cells, whereas a negative log_2_ FC is associated with up-expression of proteins in Fe(II)/HS^−^-grown cells. The bubble size for each protein represents the mean normalized spectral intensity of three biological replicates for FeS_2_-grown cells (positive log_2_ FC) or for Fe(II)/HS^−^-grown cells (negative log_2_ FC). The significance of differential expression for each protein, calculated as −log_10_(*P*), is plotted along the *y* axis, with increasing values indicating higher statistical significance. Colored bubbles indicate proteins with significant (*P < *0.05) differential expression with a log_2_ FC of at least 1. Dashed lines show cutoff values for significance and log_2_ FC.

Using informatics approaches (61), we next identified proteins with putative [Fe-S] cluster binding domains, as these proteins could be involved in assimilating and/or trafficking the putative reductive dissolution product of FeS_2_ reduction, FeS_aq_. A total of 101 proteins with [Fe-S]-binding motifs were predicted in the M. voltae proteome. Of these, 52 were significantly differentially expressed between FeS_2_-grown and HS^−^/Fe(II)-grown cells (*P < *0.05; log_2_ fold change [FC] > 1), half of which were up-expressed on FeS_2_ (Fig. 6; Data Set S1). Interestingly, a putative redox-active disulfide protein (*Mvol*_1251), the 19th most highly detected protein in the entire FeS_2_ proteome, was significantly up-expressed in the FeS_2_ growth condition (FC, 8.45; *P* < 0.01). This protein is uncharacterized but shows homology to putative archaeal thioredoxins. Further, six of the 17 putative [4Fe-4S] ferredoxin binding domain proteins encoded in the M. voltae genome were significantly up-expressed in the FeS_2_-grown cells, while only two were up-expressed in Fe(II)/HS^−^-grown cells; the remaining nine were similarly expressed between the two growth conditions. One of these proteins (*Mvol*_0876) is homologous to formylmethanofuran dehydrogenase, subunit G (FwdG) and is found in a gene cluster with other Fwd-encoding genes. In addition, two of these proteins (*Mvol*_1602 and *Mvol*_1603) are homologous to subunits of the membrane-bound hydrogenase (Eha), EhaP, and EhaQ, respectively. Inferred functions of the other three up-expressed putative ferredoxin-binding proteins (*Mvol*_0606, *Mvol*_1449, and *Mvol*_0711) could not be established based on homology to characterized proteins or on functionalities of proteins encoded by adjacent genes. The genome of M. voltae encodes two rubredoxin proteins, both of which were significantly up-expressed on FeS_2_, supporting our observation of features consistent with rubredoxin in EPR spectra of FeS_2_-grown cells (Fig. 6; Fig. S5). Two aldo-/ketoreductases are encoded in the M. voltae genome, and both were significantly up-expressed on FeS_2_ as well. The aldo-/ketoreductases are currently being evaluated for their potential role(s) in supplying reducing equivalents for the reduction of FeS_2_.

We next examined the abundance of proteins that might be involved in Fe transport in M. voltae using informatics approaches. In bacteria, the Feo system functions to transport Fe(II) into the cell (62, 63). The transmembrane protein, FeoB, is generally accompanied by a soluble component, FeoA, that has a regulatory function (63). NTP-driven, FeoAB-mediated transport of Fe(II) has been suggested to be the primary mechanism of incorporating Fe in methanogens based on informatics data (64). The proteome of M. voltae contains two copies of FeoA (*Mvol*_0977 and *Mvol*_0619), both of which were significantly up-expressed in cells grown with FeS_2_ relative to those grown with Fe(II)/HS^−^. Adjacent to the more highly expressed FeoA gene is a gene encoding FeoB (*Mvol*_0975), which was also significantly up-expressed when cells were grown on FeS_2_ compared to Fe(II)/HS^−^ (Data Set S1). Similarly, a homolog of DtxR (*Mvol*_0620*)*, a negative transcriptional regulator involved in maintaining transition metal homeostasis in *Bacteria* and some *Archaea* (65–67), was up-expressed in FeS_2_- versus Fe(II)/HS^−^-grown cells. When Fe(II) is limiting and is unavailable, DtxR is inactive. Conversely, when DtxR binds available Fe(II), it suppresses genes involved in Fe uptake and transport, such as FeoAB, to maintain cytoplasmic Fe homeostasis (65–67). Increased expression of DtxR and FeoAB could imply that the cells incorrectly sense Fe(II) limitation when grown with FeS_2_ compared to those grown with Fe(II)/HS^−^. This is potentially consistent with the smaller size of cells grown with FeS_2_ compared to those on Fe(II)/HS^−^ (Fig. 1a), possibly attributable to perceived nutrient (i.e., Fe) limitation. However, FeS_2_-grown cells accumulate 193% more Fe per unit cell volume than Fe(II)/HS^−^-grown cells (Fig. 1b), and this extra Fe is possibly stored as a thioferrate-like phase, based on EPR data (Fig. 3 and 4). Given that cells were growing at the same rate and were in the same stage of growth when they were harvested for analyses, as shown in growth curves reported in a recently published article (26), these differences are attributed to the different Fe and S sources used to cultivate cells.

The apparent contradiction between sensed iron limitation and iron hyperaccumulation (in an IssA-type, mineralized context and/or an abundance of [Fe-S]-binding proteins) may be less a consequence of the intracellular availability of Fe than of S, under the growth conditions used, considering that cells need more S than they do Fe (9). We hypothesize that to simultaneously meet Fe and S demands, cells passively assimilate uncharged FeS_aq_ or FeSH^+^, facilitated via an unknown transporter. FeS_aq_ could be generated either through dissolution of FeS_mack_ or Fe_1−_*_x_*S_pyr_ that has reductively precipitated onto the surface of FeS_2_ (27), or from spontaneous association of Fe(II) and HS^−^ to form FeS_aq_ when HS^−^ concentrations exceed >1 μM (15), as in the experiments outlined herein. Passive assimilation would occur along the concentration gradients for Fe and S, which would be expected to favor uptake of FeS into the cell. Further metabolism of the Fe and S once inside the cell may favor immediate deposition into storage formats like IssA-thioferrate when intracellular S availability is low relative to Fe, particularly if intracellular trafficking and usage of Fe(II) occur in conjunction with HS^−^ or thiolate ligands. Alternatively, resorption of Fe out of a mineralized storage phase may obligately require the participation of S species. Further investigations into the role of the DtxR/FeoAB system(s) in Fe transport and regulation and possible coregulation of S metabolic pathways are needed to evaluate these hypotheses.

The observation of similar levels of expression of proteins involved in core methanogenesis pathways and similar (note exceptions above) levels of expression of putative [Fe-S] binding proteins in the proteomes of M. voltae grown with Fe(II)/HS^−^ versus those grown with FeS_2_ implies that the pathways of trafficking Fe/S in these cells are largely similar. Little is known of how Fe and S are trafficked in methanogens, although informatics analyses reveal that the only known [Fe-S] cluster biosynthesis pathway in these cells is the sulfur pathway (SUF) (8, 68). Originally described in Escherichia coli (69), the full SUF system is composed of the genes *sufABCDSE* (70, 71). However, the genome of M. voltae encodes homologs of only a scaffold protein, SufB (*Mvol*_0653), and the ATPase SufC (*Mvol*_0654) and lacks homologs of cysteine desulfurase (SufS) (8), which, in E. coli and other cells (72), donates S from cysteine to SufBC for [Fe-S] cluster biosynthesis. This lack of a SufS homolog is consistent with the inability of M. voltae to use cysteine as a S source for growth (73).

In methanogens, cysteine is synthesized from HS^−^ (9) via the tRNA-dependent SepRS/SepCysS pathway (74). First, tRNACys is aminoacylated with *O*-phosphoserine (Sep) via the activity of *O*-phosphoseryl-tRNA synthetase (SepRS). Next, Sep-tRNACys is converted to CystRNACys by Sep-tRNA:Cys-tRNA synthase (SepCysS). M. voltae encodes homologs of both enzymes (*Mvol*_0433 and *Mvol*_0651, respectively). To gauge if M. voltae alters how it processes/traffics Fe and S for [Fe-S] cluster and cysteine biosynthesis using the SUF and SepRS/SepCysS pathways, respectively, when grown on FeS_2_ versus Fe(II)/HS^−^, we compared the profiles of the peptides from each pathway. No significant differences in expression of SufBC were detected in cells grown on either source of Fe and S. While we detected over 75% of proteins from the predicted proteome of M. voltae, we did not capture peptides that match SepRS. However, SepCysS was detected under both growth conditions at similar levels. Thus, M. voltae does not appear to alter the expression of either the SUF or SepRS/SepCysS pathways in response to growth on FeS_2_.

We next examined the genome of M. voltae for proteins with homology to the IssA homolog identified in P. furiosus that coordinates thioferrate as a mechanism to store Fe and S. Specifically, we examined the genome for protein homologs that encode the N-terminal 1 to 109 residues (IPR003731 domain) of P. furiosus IssA, since this domain has been overexpressed, purified, and biochemically shown to be capable of binding 25 equivalents of Fe per monomer (58). Two proteins encoded in the M. voltae genome (*Mvol*_0689 and *Mvol*_0693) exhibited homology (25% and 27% sequence identities) to the N-terminal domain of P. furiosus IssA, and both were upregulated under the FeS_2_ growth condition (Data Set S1). The up-expression of *Mvol*_0689 and *Mvol*_0693 gene products in FeS_2_- relative to Fe(II)/HS^−^-grown cells is possibly consistent with a role in Fe/S metabolism, since this is the only variable that differs in this treatment. As mentioned above, it is possible that one or both homologs function to store or sequester thioferrate-like nanoparticles during growth with FeS_2_. Alternatively, given the unique temperature relaxation profile for the broad signal in M. voltae cells tentatively assigned to thioferrate-like particles (Fig. 4), it is possible that these gene products or other unknown proteins act to stabilize different forms of Fe/S-based nanoparticles. To further assess this possibility, we examined TEM images of M. voltae cells grown with Fe(II)/HS^−^ or FeS_2_. A greater abundance of electron-dense regions was observed in FeS_2_-grown cells that could be attributable to Fe-containing nanoparticles in the cytoplasm of the cells. However, similar electron dense regions were also observed in Fe(II)/HS^−^ grown cells, albeit to a lesser extent (Fig. S1). Additional work will be necessary to determine whether IssA homologs in M. voltae can coordinate Fe/S nanoparticles, to determine the form of Fe/S that is stored/sequestered in these nanoparticles, and to determine whether Fe/S storage/sequestration as nanoparticles represents a mechanism for cells to maintain Fe homeostasis.

### Conclusions.

M. voltae exhibits clear phenotypic differences in response to growth with Fe(II)/HS^−^ and with FeS_2_. Distinct differences in cell morphology were observed between growth conditions, most notably the significantly reduced size of cells growing with FeS_2_ compared to those grown with Fe(II)/HS^−^. While the cells are smaller, it is not necessarily due to an effect of Fe limitation, as the cells from the FeS_2_ growth conditions were found to have more Fe on a per-cell volumetric basis than those grown with Fe(II)/HS^−^. Differential proteomics analyses suggest little if any difference in the energetic state of cells grown with FeS_2_ versus Fe(II)/HS^−^, as indicated by similar abundances of proteins involved in the core energy metabolism pathways of these cells. Differential proteomics analyses and whole-cell EPR identified some difference in the expression and deployment of [Fe-S] clusters among the proteomes of FeS_2_- versus Fe(II)/HS^−^-grown cells, including in small putative ferredoxin and rubredoxin domain proteins, as well as several oxidoreductases (e.g., aldo-/ketoreductases and redox-active disulfide protein) that are currently being evaluated for their potential roles in supplying reducing equivalents for the reduction of FeS_2_.

A broad EPR signal observed in FeS_2_-grown cells but not in Fe(II)/HS^−^-grown cells, combined with proteomics data indicating up-expression of two homologs of IssA in M. voltae and the observation of electron-dense regions in cells growing with FeS_2_, suggests that M. voltae may store Fe/S as thioferrate-like nanoparticles, similar to what has been described for P. furiosus (58). However, the temperature dependence of the broad EPR signal observed for M. voltae cells grown with FeS_2_ suggest that the Fe/S phase is likely distinct from the thioferrate phase previously detected in P. furiosus cells. Nonetheless, these combined observations indicate that M. voltae cells accumulate Fe when grown on FeS_2_ relative to Fe(II)/HS^−^. Paradoxically, differential proteomics analyses revealed that two homologs of FeoA and a single homolog of FeoB putatively involved in transport of Fe(II) and a transcriptional regulator (DtxR) putatively involved in sensing Fe(II) and regulating Fe homeostasis were upregulated in FeS_2_- versus Fe(II)/HS^−^-grown cells. This indicates that cells incorrectly sense Fe limitation when grown with FeS_2_. These observations could suggest that the intracellular metabolism of the stored Fe depends on the availability of excess S species that either intercept the Fe prior to deposition into storage or are necessary for its retrieval from the stored phase.

We put forward a model for Fe/S acquisition, deployment, and storage/sequestration aimed at reconciling observations that collectively point to cells apparently sensing and responding to Fe limitation while at the same time pointing to enhanced Fe storage/sequestration in FeS_2_-grown cells, relative to Fe(II)/HS^−^-grown cells. Following cell-mediated reduction of FeS_2_, FeS_mack_ or Fe_1−_*_x_*S_pyr_ precipitates on the surface of FeS_2_ (27), and dissolution of FeS_mack_ or Fe_1−_*_x_*S_pyr_ in the presence of >1 μM sulfide favors FeS_aq_ cluster species as the predominant form of Fe(II) in solution (15). Such species could be scavenged and transported into the cell in protein-bound states. Alternatively, one could envision direct transport of FeS^0^ or FeSH^+^ via as-yet-unidentified transporters. Either situation could lead to excess Fe (and possibly S) in the cell that is stored/sequestered as thioferrate-like nanoparticles, since the Fe and S are assimilated in an ∼1:1 ratio and cells require more S than Fe (9). In this model, DtxR, which binds Fe(II) under Fe-replete conditions and downregulates expression of proteins involved in Fe assimilation, such as FeoAB (65–67), would not sense Fe(II), since it is present primarily as FeS_aq_ and/or protein-bound FeS species. The lack of Fe(II) binding to DtxR would lead to accumulation of Fe in the cell that is stored or sequestered by IssA-like homologs as thioferrate-like species, as mechanism to limit Fe toxicity. This model provides a framework for the rational design of targeted physiological, biochemical, and genetic experiments aimed at further characterizing Fe acquisition and homeostasis in M. voltae, with possible application to other methanogens.

## MATERIALS AND METHODS

### Preparation of synthetic nanoparticulate pyrite.

All chemicals used in mineral synthesis were American Chemical Society grade or higher. All glassware was first washed in 10% nitric acid and rinsed three times with 18.2-Ω Milli-Q water (MQ H_2_O). Pyrite (FeS_2_) was synthesized according to methods outlined by Berner (16). Briefly, within an anaerobic chamber (Coy Labs, Grass Lake, MI), 14.4 g Na_2_S · 9H_2_O and 16.7 g FeSO_4_ · 7H_2_O were separately dissolved in 50 ml of anoxic MQ H_2_O. These two solutions were then combined in a 500-ml bottle and stirred vigorously for 15 min, at which point 2.1 g of elemental sulfur (S^0^) was added. The bottle was then sealed with a butyl rubber stopper, removed from the chamber, and bubbled with N_2_ passed over heated (200°C) and H_2_-reduced copper shavings for 45 min. Following purging, the solution was incubated anoxically for 4 days at 65°C and then for another 4 days at 85°C. After incubation, the FeS_2_ was washed aerobically in sealed centrifuge tubes (via centrifugation [1,000 × *g*, 10 min, 4°C] and decanting) in the following series to remove unreacted HS^−^, Fe(II), FeS, and S^0^: four rinses with 1 N HCl, one rinse with boiling 6 N HCl, two rinses with MQ H_2_O, and three rinses with >99.5% acetone. The FeS_2_ was then transferred to an anaerobic chamber and further washed three times with sterile MQ H_2_O. After washing, the FeS_2_ was pelleted via centrifugation (1,000 × *g*, 10 min, 4°C) and transferred back into an anaerobic chamber; the aqueous phase was decanted, and the pellet was resuspended in sterile, anoxic MQ H_2_O in a butyl rubber-stoppered sterile serum bottle. Finally, the headspace of the bottle was purged with 0.2-μm-filtered ultra-high-purity N_2_ gas. The percentage (wt/vol) of the slurry was determined by drying 1 ml of slurry in triplicate under N_2_ and weighing the dried product. FeS_mack_, used here in EPR experiments, was synthesized as we described previously (26).

### Strains and cultivation medium.

Methanococcus voltae strain A3 was obtained from the American Type Culture Collection (ATTC-BAA-1334). M. voltae was grown in Fe- and S-free basal medium that contained the following (in grams per liter): NaCl, 21.98; MgCl_2_ · 6H_2_O, 5.10; NaHCO_3_, 5.00; NH_4_Cl, 0.50; K_2_HPO_4,_ 0.14; KCl, 0.33; and CaCl_2_ · 2H_2_O, 0.10 (Table S3). The basal medium was amended with 0.01 g liter^−1^ Fe(NH_4_)_2_(SO_4_)_2_ · 6H_2_O and 0.48 g liter^−1^ Na_2_S · 9H_2_O for Fe(II)/HS^−^-grown cells after autoclaving. Sulfide was added from an anoxic, sterile stock solution 30 min prior to inoculation. For FeS_2_-grown cells, basal medium was amended with FeS_2_ to a final concentration of 2 mM Fe prior to inoculation. Basal medium was amended (each 1% [vol/vol]) with trace element, vitamin, formate, and organic solutions prior to inoculation. The trace element solution used was based on the work of Whitman et al. (73) and was amended to omit Fe and replace sulfate salts with chloride salts at the same molar concentrations. The trace element solution contained the following (in grams per liter): nitriloacetic acid, 1.500; MnCl_2_ · 4H_2_O, 0.085; CoCl_2_ · H_2_O, 0.100; ZnCl_2_, 0.047; CuCl_2_ · 2H_2_O, 0.0683; NiCl_2_ · 6H_2_O, 0.0683; Na_2_SeO_3_, 0.200; Na_2_MoO_4_ · 2H_2_O, 0.100; and Na_2_WO_4_ · 2H_2_O, 0.100. The vitamin solution contained (in grams per liter): pyridoxine HCl, 0.01; thiamine HCl, 0.005; riboflavin, 0.005 g; nicotinic acid, 0.005; calcium D(+) pantothenate, 0.005; biotin, 0.002; folic acid, 0.002; and cobalamin, 0.0001. The organic solution consisted of 1 M sodium acetate · 3H_2_O, 75 mM l-leucine HCl, and 75 mM l-isoleucine HCl. The formate solution contained 40% (wt/vol) sodium formate.

All basal medium components, except NaHCO_3_, were dissolved in MQ H_2_O and then boiled for 15 min. After boiling, the medium was sealed with a butyl rubber stopper and sparged with N_2_ gas passed over a heated (200°C) and reduced copper column for 1 h per liter of medium. After degassing, the medium was sealed and brought into an anaerobic chamber, and NaHCO_3_ was added as specified above. After addition of these components, the pH of the medium was adjusted to 7.00 using anoxic 2 M HCl or 1 M NaOH. For proteomic experiments, 75 ml of medium was dispensed into 165-ml serum bottles. For AA and EPR experiments, as well as experiments aimed at testing cell-mineral separation techniques, 800 ml of medium was dispensed into 2-liter medium bottles. Once dispensed, bottles were sealed with 20-mm black rubber stoppers and aluminum crimp caps (165-ml serum bottles) or with number 6.5 black rubber stoppers and sealed with screw-cap tops that had been modified by drilling to provide a sampling port to access the stopper (2-liter medium bottles). The headspace of the medium bottles was purged with 80:20 N_2_-CO_2_ for 30 min. Two-liter medium bottles were carefully vented with a 22-gauge needle to ∼1 atm and stored upside down in individual metal autoclave bins to minimize in-gassing of O_2_ from the atmosphere and to ensure the safety of personnel during autoclaving, respectively. The bottles were then autoclaved for 20 min at 123°C.

### Cultivation of M. voltae.

Cultures of M. voltae were maintained by weekly transfers (10% [vol/vol]) into fresh medium with formate as the methanogenesis substrate and Fe(II) and HS^−^ as sole Fe and S sources, respectively. Cells were washed prior to inoculation by pelleting them in sealed 50-ml tubes (Globe Scientific, Mahwah, NJ) by centrifugation (4,696 × *g*, 4°C, 20 min) in a swing-out bucket rotor. Spent medium was decanted in an anaerobic chamber, and the cell pellet was resuspended in sterile and anoxic Fe/S-free base salts medium. All experiments used washed M. voltae cells, grown with 26 μM Fe(II) and 2 mM HS^−^, as the inoculum (10% [vol/vol]). After inoculation, the headspace of microcosm assays was flushed with an 80:20 (vol/vol) mixture of N_2_-CO_2_ gas that had been passed through a 0.2-μm filter for at least 15 min. Finally, the culture bottles were pressurized to 3.21 or 1.65 atm for 165-ml and 2-liter reactors, respectively, inoculated, and incubated at 38°C in the dark and statically on their sides to minimize disruption of microbe-mineral interactions while maximizing gas diffusion.

### Measurement of activity and growth.

Headspace gas from cultures was sampled with a N_2_-flushed syringe equipped with a stopcock and needle and were diluted with ultra-high purity N_2_ into CaliBond bags (Calibrated Instruments Inc., Manhasset, NY) prior to CH_4_ determination. CH_4_ was determined by gas chromatography by injecting 5 ml of sample into an injector valve set at 55°C on a SRI 8610C gas chromatograph (SRI Instruments, Torrance, CA) equipped with a 4.5-m by 0.125-in. (outside diameter) Hayesep DB 100/120 packed column with the oven set to 44°C (Valco Instrument Company Inc., Houston, TX). CH_4_ was detected by a flame ionization detector set at 156°C with ultra-high-purity He as the carrier gas. CH_4_ peak area values were converted to parts per million using a standard curve (EGAS Depot, Nampa, ID). Dissolved HS^−^ was determined via colorimetry (670 nm) using the methylene blue assay (75) and converted to total sulfide (dissolved and gas phase) using a standard Henry’s Law solubility constant of 0.101 M·atm^−1^ for HS^−^ that was temperature (38°C) adjusted to 0.075 (76). Absorbance was measured using a Genesys 10S visible-spectrum (Vis) spectrophotometer (Thermo Fisher Scientific, Waltham, MA). Growth was determined by direct counting of cells using a Petroff-Hausser counting chamber on a Nikon YS100 light microscope with a 100× oil lens objective (Nikon, Tokyo, Japan). Prior to counting, an aliquot of cells was collected and concentrated by centrifugation at 15,000 × *g* for 15 min in a fixed-angle rotor.

### Culture harvesting and cell-mineral separation.

Large (800 ml in 2.0-liter bottles) cultures of M. voltae were used for AA and EPR analyses. Cultures were monitored for cells as described above and were harvested at mid-log phase after cultures had reached a density of >10^8^ cells ml^−1^. Biomass was concentrated anaerobically from 800 ml of cultures via centrifugation (15,000 × *g*, 4°C, 60 min) in a Sorvall RC-5B centrifuge with a fixed-angle GSA rotor (DuPont Instruments, Wilmington, DE) and 250-ml centrifuge bottles equipped with gas-tight seals (Nalgene Nunc International, Rochester, NY). After centrifugation, the bottles were transferred back to the anaerobic chamber and the supernatant was carefully decanted. The pellets in each bottle were resuspended and then recombined in 50 ml of sterile and anoxic Fe/S-free base salts medium. The 50-ml concentrate was then further centrifuged (4,696 × *g*, 4°C, 60 min) in a swing-out bucket rotor, and the supernatant was carefully decanted. The pellet was then further processed for cell separation (see below) for use in AA or EPR.

Our previous observations indicated that cells grown with FeS_2_ preferentially associate with the mineral (26). We therefore developed and tested a protocol to separate cells from FeS_2_. To achieve this, we took a pellet from an 800-ml culture and resuspended it in 8 ml of sterile and anoxic Fe/S-free base salts medium. The cell concentrate was then poured into a 15-ml centrifuge tube (Globe Scientific). Percoll (GE Healthcare, Chicago, IL) was prepared according to the manufacturer’s directions and was adjusted to 0.4 M NaCl. The Percoll working solution was then sparged with 0.2-μm-filtered N_2_ via a canula and vent needle for 45 min per 100 ml aliquot in a sealed glass serum bottle. The solution was then brought into the anaerobic chamber, and 4 ml was slowly underlaid beneath the cell concentrate by addition via a sterile cannula and syringe. Cells were first separated from bulk mineral by a slow spin at 1,000 × *g* for 10 min at 4°C in a swing-out bucket rotor. The samples were then removed and returned to the anaerobic chamber. A pipette was then used to extract the cell fraction that overlay the Percoll (∼8.5 ml of cell extract and minor amounts of Percoll) and was transferred to a fresh 50-ml centrifuge tube. Sterile and anoxic Fe/S-free base salts medium was then added to increase the volume to 40 ml. The tubes and their contents were mixed by mild vortexing, and the cells were concentrated by centrifugation (4,696 × *g*, 4°C, 30 min). In the anaerobic chamber, the supernatant was carefully decanted, and the cells were again resuspended in 8 ml of sterile and anoxic Fe/S-free base salts medium and transferred to a 15-ml centrifuge tube. At this point, 1 ml of an anoxic, filter-sterilized detergent solution containing 0.02% Tween 80, 0.4 M NaCl, 20% methanol, 20 mM EDTA, and 20 mM sodium pyrophosphate was added (modified from reference 77). The sample was then vortexed for 10 s to mildly disrupt any cell/mineral interactions while attempting to keep whole cells intact. As before, 4 ml of Percoll working solution was added under the sample, and the cells were subjected to a second round of centrifugation (2,000 × *g*, 4°C, 20 min). The cells were extracted with a pipette, transferred to a clean 50-ml centrifuge tube, and washed in 40 ml of basal medium. The resulting cell pellet was then used for AA and EPR spectroscopic analyses (see below).

The efficacy of the cell separation protocol was examined using 800 ml culture of M. voltae grown on Fe(II)/HS^−^. Cell biomass was concentrated by centrifugation, as described above, and the cell pellet was resuspended in 9 ml of anoxic base salts medium. Triplicate 3-ml aliquots of the cells were dispensed into 15-ml centrifuge tubes containing 1 ml of base salts medium (control) or 1 ml base salts medium amended with FeS_2_. The amount of FeS_2_ added was the amount that would have been present in a culture of equivalent volume. The volume of each sample was then increased to 8 ml using anoxic base salts medium. These samples were then separated from the added minerals as described above, and the cell density of the extract was determined via direct counting as described above. The remainder of the cells were pelleted and processed for AA analysis (see below).

### Sample preparation for AA and EPR spectroscopy.

Washed cell pellets from 800 ml cultures were resuspended in 2.5 ml of anoxic base salts medium using a pipette. Once pellets were fully resuspended, 2,000 μl, 500 μl, and 10 μl of the cell suspension were subsampled for EPR spectroscopy, AA spectroscopy, and cell density determination, respectively. Samples for EPR and AA spectroscopic analyses were placed into 2.0- or 1.5-ml screw-cap microcentrifuge tubes with O-ring fittings, respectively (Thermo Fisher Scientific). The tubes and their contents were then centrifuged in a fixed-angle rotor at 15,000 × *g* for 15 min at 4°C. The samples for cell counts were diluted 40-fold and subjected to cell counting immediately. After centrifugation, the supernatant of the samples for use in EPR and AA spectroscopy was removed from the cell pellet using a pipette. Cell pellets for determination of Fe content via AA spectroscopy were stored at −80°C, while those for EPR spectroscopy were resuspended in 300 μl of anoxic base salts medium containing 25% glycerol as a glassing agent. Samples for EPR spectroscopy were transferred into EPR tubes (4-mm OD; Wilmad Lab Glass, NJ, USA), capped with rubber septa, and then immediately removed from the chamber and flash frozen in liquid N_2_. Tubes and their contents were stored at liquid N_2_ temperatures until spectral acquisition occurred.

### AA spectroscopy.

Frozen cell pellets for AA spectroscopic analysis were thawed at room temperature (∼21°C), and then 750 μl of 10% trace metal-free nitric acid was added to the samples. The tubes were sealed, and the pellets were resuspended by gentle pipetting and mild vortexing. The tubes were then incubated for 36 h at 98°C in a Isotemp heat block (Thermo Fisher Scientific). The tubes were checked approximately every 12 h and were vortexed to encourage digestion. Once the samples had fully digested, the samples were diluted with 0.75 ml MQ H_2_O and were again mixed by mild vortexing. Using a 0.2-μm PTFE syringe filter that had been prerinsed with 5 ml of MQ H_2_O, the samples were filtered into sterile 2.0-ml microcentrifuge tubes. The samples were then analyzed by flame AA spectroscopy using an Agilent 240 FS instrument (Agilent Technologies Inc., Santa Clara, CA) equipped with an Fe lamp utilizing an acetylene/air mixture (11:60 lb/in^2^ partial pressures) as a fuel source.

The Fe content for the samples was determined using a standard curve prepared from a 1,000-ppm Fe standard (Ricca Chemical Company, Arlington, TX). All dilutions and standards were prepared in fresh 5% nitric acid. The Fe content for each sample was then used to determine the amount of Fe on a per-cell basis, which was ultimately normalized to cell volume as determined with TEM.

### EPR spectroscopy.

Low-temperature, continuous-wave (CW), X-band (9.38 GHz) EPR spectra were collected using a Bruker EMX spectrometer fitted with a ColdEdge (Sumitomo Cryogenics) 10 K waveguide in-cavity cryogen-free system, a helium Stinger recirculating unit (Sumitomo Cryogenics, ColdEdge Technologies, Allentown, PA), and an Oxford Mercury iTC controller unit. For measurements below 75 K, helium gas flow was maintained at 100 lb/in^2^; higher-temperature measurements required decreased supply pressure. Unless otherwise noted, the typical spectral parameters were as follows: microwave power, 5.3 mW; modulation frequency, 100 kHz; and modulation amplitude, 10 G. All spectral data were plotted in the OriginPro software program (2018b; OriginLab Corp. Northampton, MA, USA). EPR spin quantitation was accomplished via double integration of *S* = 1/2 [Fe-S] cluster signals and comparison to a 100 μM copper(II) triethanolamine spin standard under nonsaturating conditions, according to the method of Aasa and Vänngård (78).

### Protein extraction, quantification, and digestion.

Prior to extracting protein from samples, a control experiment was conducted to determine potential effects of FeS_2_ on protein extraction and recovery. An 800-ml culture of M. voltae grown with Fe(II)/HS^−^ was concentrated via centrifugation as described above. The concentrated pellet was resuspended in 7 ml of anoxic base salts medium, and six 1.0-ml aliquots were dispersed into sterile 2.0-ml microcentrifuge tubes. Three tubes were amended with 0.0175 g FeS_2_ and three received no added FeS_2_. This is the amount of FeS_2_ that would be added to a 75-ml culture. The tubes were mixed by inversion and 30 s of mild vortexing and were then subjected to centrifugation (15,000 × *g*, 4°C, 15 min). The supernatant was carefully discarded, and the cell pellet and any added FeS_2_ were frozen at −80°C until analysis. The cells were then digested as described above, 40 μg of protein extract was analyzed by SDS-PAGE, and a subsample was quantified with a NanoDrop spectrophotometer (Thermo Scientific, San Jose, CA). For SDS-PAGE analyses, aliquots of protein were combined with Laemmli buffer (Bio-Rad, Hercules, CA), and 40 μg of protein was loaded to each lane of a 4 to 20% Mini-Protean TGX precast gel (Bio-Rad). Gel electrophoresis was performed at 150 V for approximately 45 min using SDS buffer. There was no discernible difference in the amount of protein recovered (Bradford assay and NanoDrop spectrometry) or the banding pattern of proteins on an SDS-PAGE gel in cell pellets incubated in the presence or absence of FeS_2_ (Fig. S7). Thus, steps were not used to remove FeS_2_ from cultures prior to subsequent protein extraction for proteomics analyses.

For proteomics analyses, 75-ml cultures of M. voltae were grown in 165-ml serum bottles that were harvested during mid-log phase of growth. The entirety of the cultures was harvested anaerobically within an anaerobic chamber by transferring cultures into 50-ml centrifuge tubes (Globe Scientific) that were centrifuged at 4,696 × *g* for 20 min at 4°C in a swing-out bucket rotor. Pellets from the same sample were combined and then recentrifuged as above. The supernatant was then carefully decanted, and the pellets were immediately placed at −80°C.

Phosphate-buffered saline (PBS [pH 7.0]; 137 mM NaCl, 2.7 mM KCl, 10 mM Na_2_HPO_4_, and 1.8 mM KH_2_PO_4_) containing protease inhibitor (complete mini-EDTA-free protease inhibitor cocktail; Roche) was used to resuspend cell pellets for protein extraction. The cells were suspended in PBS, placed on ice, and sonicated for 15 min using a Biologix ultrasonic homogenizer 3000 using 10 pulses at 100 W and 2 kHz each for 3 s, after which samples were subjected to centrifugation (10,000 × *g* for 30 min at 4°C) to pellet cell debris. Prechilled (−80°C) acetone was added to the samples, which were then incubated at −80°C for 1 h and then at −20°C for 12 h to precipitate protein. Samples were centrifuged to pellet protein at 10,000 × *g* for 20 min at 4°C. The supernatant was carefully decanted, leaving the protein pellet, which was stored at −80°C until subjected to digestion.

Protein digestion was performed at the University of Nevada, Reno Proteomics Center using the EasyPep mini-MS sample prep kit (Thermo Scientific, San Jose, CA). During the digestion, the samples were briefly subjected to reduction and alkylation using iodoacetamide, and then samples were digested using a trypsin-LysC mixture (modified from the work of Lundby et al. [79]). Samples were passed over a C_18_ reverse-phase column prior to liquid chromatography-mass spectrometry (LC-MS) to remove undigested protein. Protein digests were separated by LC with an UltiMate 3000 RSLCnano system (Thermo Scientific) with a self-packed ReproSil-Pur C_18_ column (100 μm by 35 cm). The column was packed at 9,000 lb/in^2^ using a nano-LC column packing kit (nanoLCMS; Gold River, CA). Chromatography was performed using a 92-min method with a 2-to-90% gradient of solvent B (0.1% formic acid in acetonitrile) and solvent A (0.1% formic acid in water). Using a digital Pico View nanospray source (New Objectives, Woburn, MA), the LC was coupled to the MS, which had an ABIRD background suppressor (ESI Source Solutions, Woburn, MA). Data-independent acquisition and MS analysis were performed using an Orbitrap Fusion MS (Thermo Scientific). To generate a reference library, 6 gas phase fractions (GPF) of the biological samples were pooled. Acquisition was performed using 4 *m/z* precursor isolation windows in a staggered pattern (GPF1, 398.4 to 502.5 *m/z*; GPF2, 498.5 to 602.5 *m/z*; GPF3, 598.5 to 702.6 *m/z*; GPF4, 698.6 to 802.6 *m/z*; GPF5, 798.6 to 902.7 *m/z*; GPF6, 898.7 to 1,002.7 *m/z*). Individual biological samples were run on the same gradient as the GPFs using a staggered window scheme (4 *m/z* Exploris 480; 24 *m/z* Fusion) and mass range of 385 to 1,015 *m/z*.

### Proteomics data and statistical analysis.

Protein fragments and retention times were identified with ScaffoldDIA (2.1.0). An empirically corrected library combining GPF and the deep neural network Prosit (80) were used to generate predicted fragments and retention times of peptides (Proteome Software, Portland, OR). Using ScaffoldDIA (2.1.0), data files were converted to mzML file format (81) using ProteoWizard (3.0.19254), and then staggered window deconvolution and alignment based on retention times were performed. The data were then individually searched against the empirically corrected library built from UniProt and Scaffold using a peptide mass tolerance of 10.0 ppm and a fragment mass tolerance of 10.0 ppm. Variable carbamidomethylation modifications to cysteine were considered. A maximum of 1 missed cleavage site by the trypsin enzyme served as the cutoff for peptide matching. Only peptides with 2 to 3 charges and 6 to 30 amino acids in length were considered. Identified peptides were assigned posterior error probabilities and filtered by Percolator (3.01.nightly-13-655e4c7-dirty) (82–84) to achieve a maximum false discovery rate (FDR) of 0.01. Quantification of peptides using Encyclopedia (0.9.2) was performed by selecting the 5 highest-quality fragment ions. To satisfy the principles of parsimony, proteins that contained similar peptides and could not be distinguished based on LC-MS analysis were assumed to be a single protein and were combined. Proteins were identified with at least 2 peptides, which achieved a protein FDR threshold of 1.0%.

Individual protein intensities returned from ScaffoldDIA (2.1.0) were log_10_ transformed and normalized using the Scaffold method. *t* tests were performed, and fold changes were calculated for differential comparisons of proteins after sum normalization using Metabolanalyst (R version 3.6.3) (85). [Fe-S]-binding proteins were predicted from the M. voltae strain A3 proteome (downloaded from UniProt on 20 July 2020) using the online server MetalPredator (61).

### Transmission electron microscopy.

Cells of M. voltae were grown to mid-log phase in 2-liter reactors with either Fe(II)/HS^−^ or FeS_2_, as described above. The cells were then subjected to the cell separation procedure described above; however, no detergent was added to samples in an attempt to maintain cell surface integrity. After the separation procedure, cells were fixed overnight (∼12 h) at 4°C in 2.5% EM-grade glutaraldehyde (Electron Microscopy Sciences, Hatfield, PA) in 0.1 M potassium sodium phosphate buffer. After fixation, the cells were washed three times in MQ H_2_O and then incubated with 2% osmium tetroxide for 4 h at room temperature (∼21°C). The fixed cells were then rinsed twice with MQ H_2_O and subjected to an ethanol dehydration series (50% to 100% molecular grade ethanol). After dehydration, the fixed and dehydrated cells were incubated twice for 10 min each in 100% propylene oxide (PO). The cells were then incubated overnight (∼12 h) at 4°C with 2:1 PO to Spurr’s embedding resin mixture. This process was repeated with cells suspended in 1:2 PO to Spurr’s, followed by 100% Spurr’s. Prior to loading into BEEM capsules (Ted Pella Inc., Redding, CA), cells in Spurr’s were brought to room temperature (∼21°C) and then centrifuged briefly to concentrate the cells. The concentrated cell pellet was transferred to a BEEM capsule and cured at 70°C overnight (∼12 h) before thin sectioning. BEEM capsules were then removed, and the sample was thin sectioned with a diamond knife on a Reichert ultramicrotome (Leica Microsystems Inc., Buffalo Grove, IL). Sections close to 60 nm thick were placed on 300 mesh copper grids (Ted Pella) and then stained with uranyl acetate and Reynold’s lead citrate. Grids were viewed with a LEO 912 transmission electron microscope (Zeiss, Oberkochen, Germany) operated at an accelerating voltage of 100 kV, and pictures were taken with a 2k-by-2k Proscan camera. Perpendicular measurements of cell diameter were manually performed on the instrument.

### Data availability.

Raw and processed proteome data have been deposited in the ProteomeXchange Consortium via the PRIDE (86) partner repository with the data set identifier PXD024933.
