# Supplementary material for: Examining Pathways of Iron and Sulfur Acquisition, Trafficking, Deployment, and Storage in Mineral-Grown Methanogen Cells
Source: J Bacteriol. 2021 Sep 8;203(19):e00146-21. doi: 10.1128/JB.00146-21 (PMC8516115; doi:10.1128/JB.00146-21)
Supplement: Supplemental file 1 — Fig. S1 to S8 and Tables S1 to S3. Download JB.00146-21-s0001.pdf, PDF file, 0.7 MB [file jb.00146-21-s0001.pdf]

Examining pathways of iron and sulfur acquisition, trafficking, deployment, and storage in mineral grown methanogen cells

Supplementary Online Information

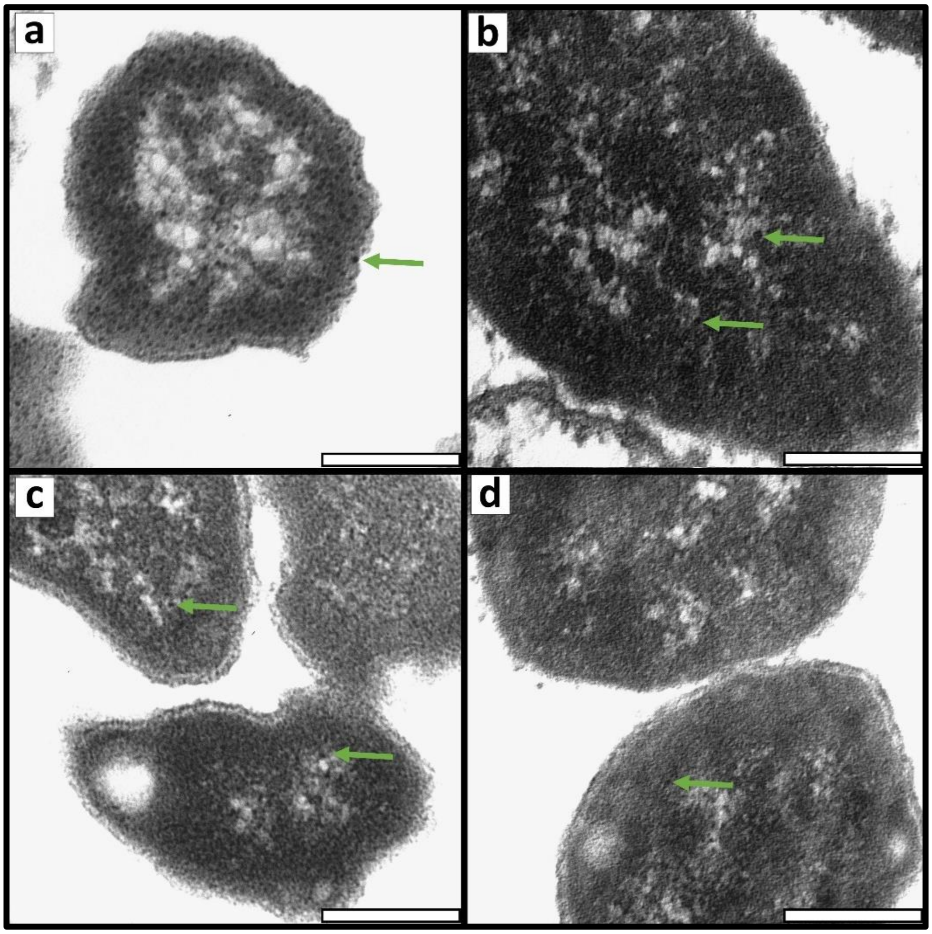

Figure S1. **Transmission electron micrographs (TEMs) of *Methanoccoccus voltae grown on different iron (Fe) and sulfur (S) sources***. *M. voltae* was grown with formate in defined medium with either synthetic nanoparticulate pyrite (**a, c**) or ferrous iron and sulfide (**b, d**) as the sole provided Fe and S sources. Cells were separated from minerals prior to TEM sample preparation with no detergent added in attempt to preserve the cell surface (see Materials and Methods). Arrows indicate electron-dense intracellular inclusions. Scale bar in all panels = 200 nm.

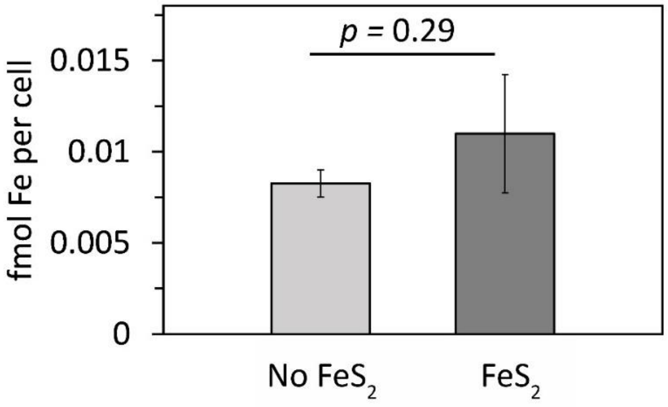

Figure S2. Iron (Fe) content per *Methanococcus voltae* cell grown with ferrous iron (Fe(II)) and sulfide (HS^-) after incubation with pyrite (FeS_2) or without FeS_2, and subjected to mineral-cell separation. *Methanococcus voltae* was grown with Fe(II) and HS^- to mid-log phase and the culture was then concentrated. The concentrated cells were then exposed to either FeS_2 or no added FeS_2 for one hour before being subjected to a mineral-cell separation protocol (see Materials and Methods). The abundance of cells following separation was then quantified before acid digestion and total iron determination via atomic absorption spectroscopy. The data represent the mean and standard deviation of three replicate incubations and Fe analyses. The result of a two-tailed Student's t-test is indicated.

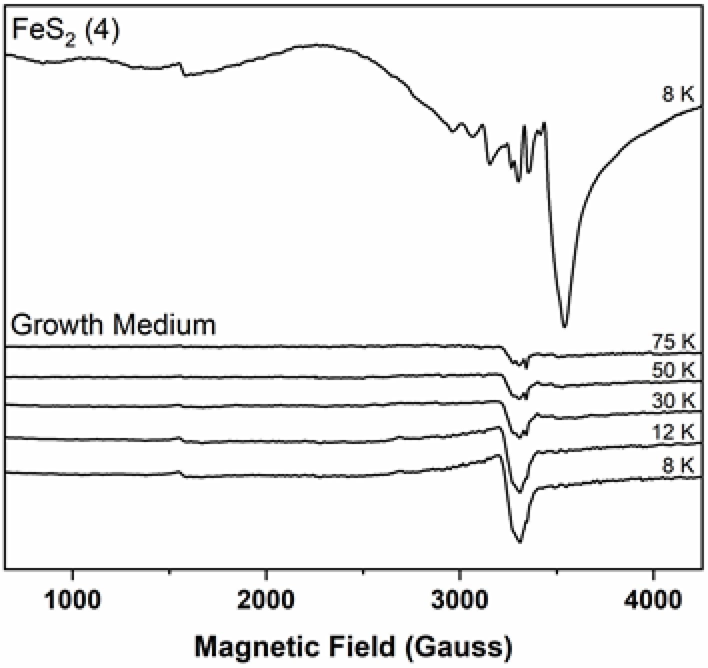

Figure S3. **Electron paramagnetic resonance data illustrating the background signal that persists in the growth medium.** All data were collected at 5 mW microwave power at the temperature values denoted. Base salts medium with 25% glycerol was prepared identically to what was used for cell samples. The FeS_2 whole cell sample (preparation 4; **Table S1**) is shown for signal intensity comparative purposes.

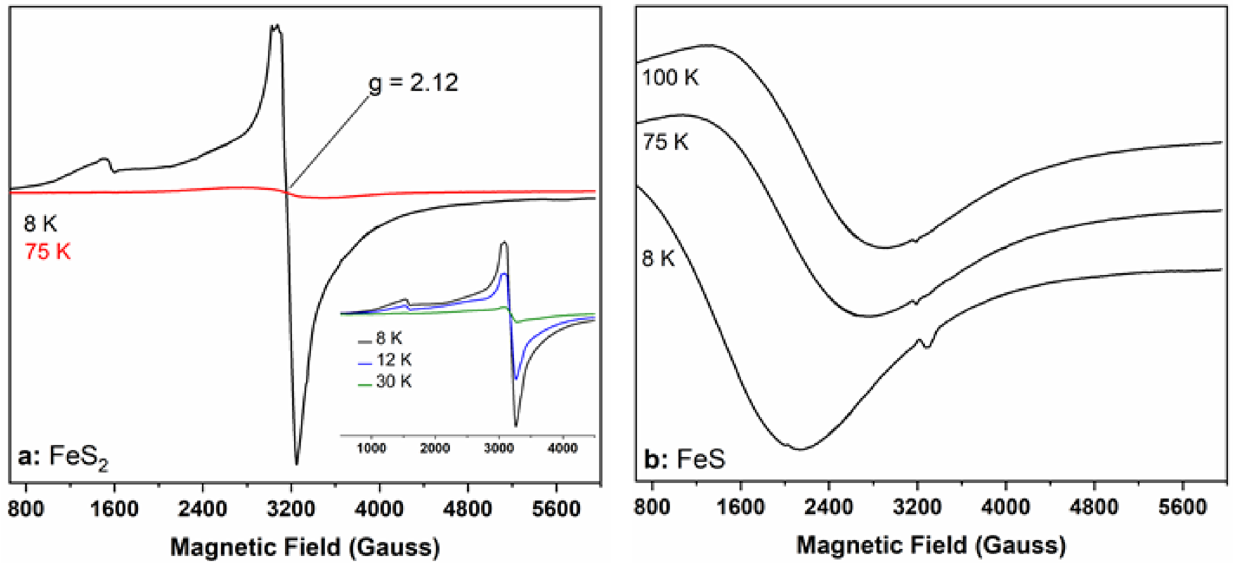

Figure S4. Electron paramagnetic resonance (EPR) spectra for synthetic nanoparticulate pyrite (FeS_2; a) and synthetic nanoparticulate mackinawite (FeS; b) in mineral growth medium used to cultivate *Methanococcus voltae*. Synthetic FeS_2 (0.10 g) or FeS_{mack} (0.07 g) were separately added to 300 µL growth medium and EPR spectra were collected. The data in both panels were recorded at 5 mW microwave power at the listed temperature values, although the inset in panel **(a)** shows data recorded at 1 mW power. FeS_2 exhibits broad paramagnetism with a distinct g value at 2.12 that exhibits fast temperature relaxation behavior. FeS exhibits broad, ill-defined paramagnetism that shows slow temperature relaxation behavior. The unique spectral features that persist in these samples do not appear to be present in any whole cell samples, providing additional support for the efficacy of our mineral-cell separation protocol.

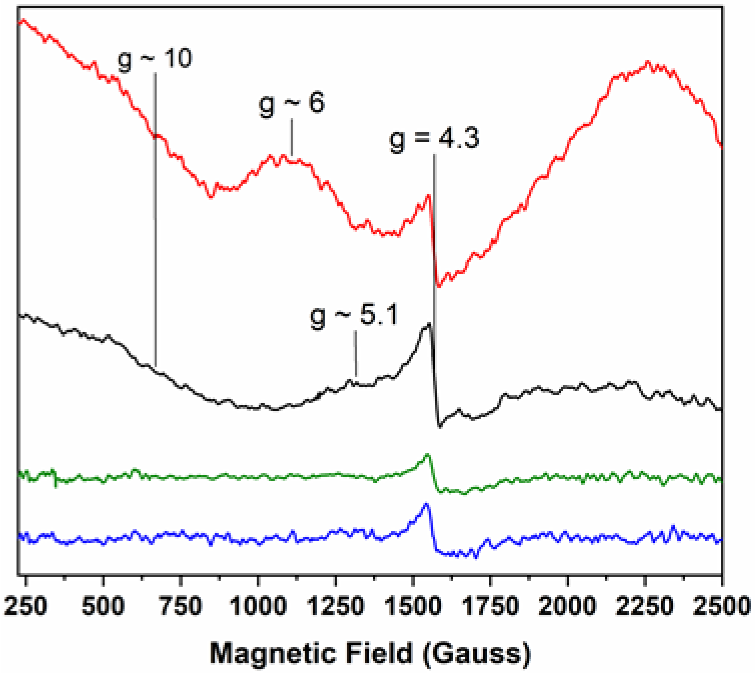

Figure S5. Electron paramagnetic resonance (EPR) spectra of whole cell *Methanoccocus voltae* whole cells grown with synthetic nanoparticulate pyrite (preparation 4, Table S1; red spectra) or ferrous iron and sulfide (preparation 3, Table S1; black spectra). The background contribution from mineral growth medium (green spectrum) and the cavity (blue spectrum; MilliQ-water sample) are also shown. All data were recorded at 8 K and 5 mW microwave power. Spectroscopic features in the g~10 region are consistent with rubredoxin-like species, while signals with g-values spanning the 4-6 region are consistent with S=3/2 $[4Fe\text{-}4S]^{+}$ clusters. The position at g=4.3 marks contributions from high spin (S=5/2) Fe(III).

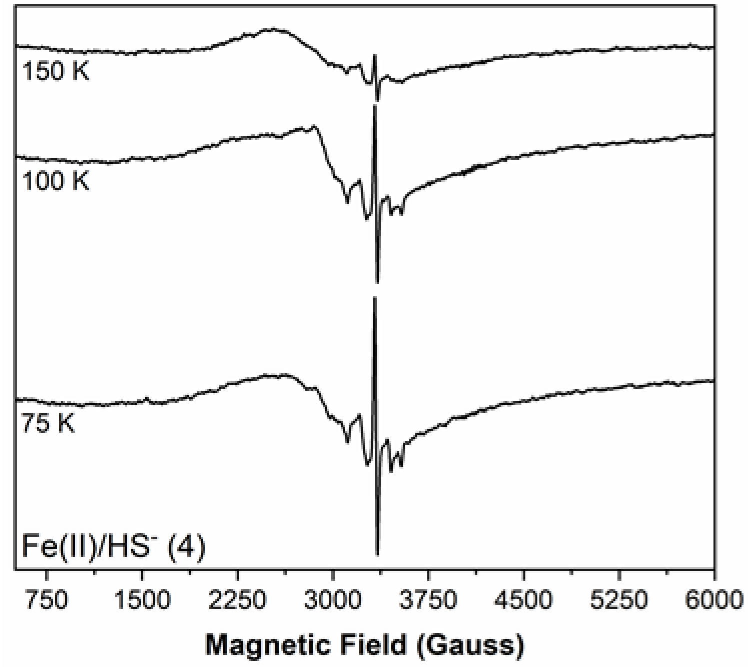

Figure S6. Electron paramagnetic resonance (EPR) spectra from *Methanoccocus voltae* a whole cells grown with ferrous iron and sulfide that exhibits a low intensity thioferrate-like feature. EPR spectra were collected at 5 mW microwave power at variable temperatures. The appearance of a low intensity, broad signal with similar line shape as observed in FeS_2-grown samples (see main body, **Figure 3**) is consistent with a small amount of thioferrate-like species in this particular sample.

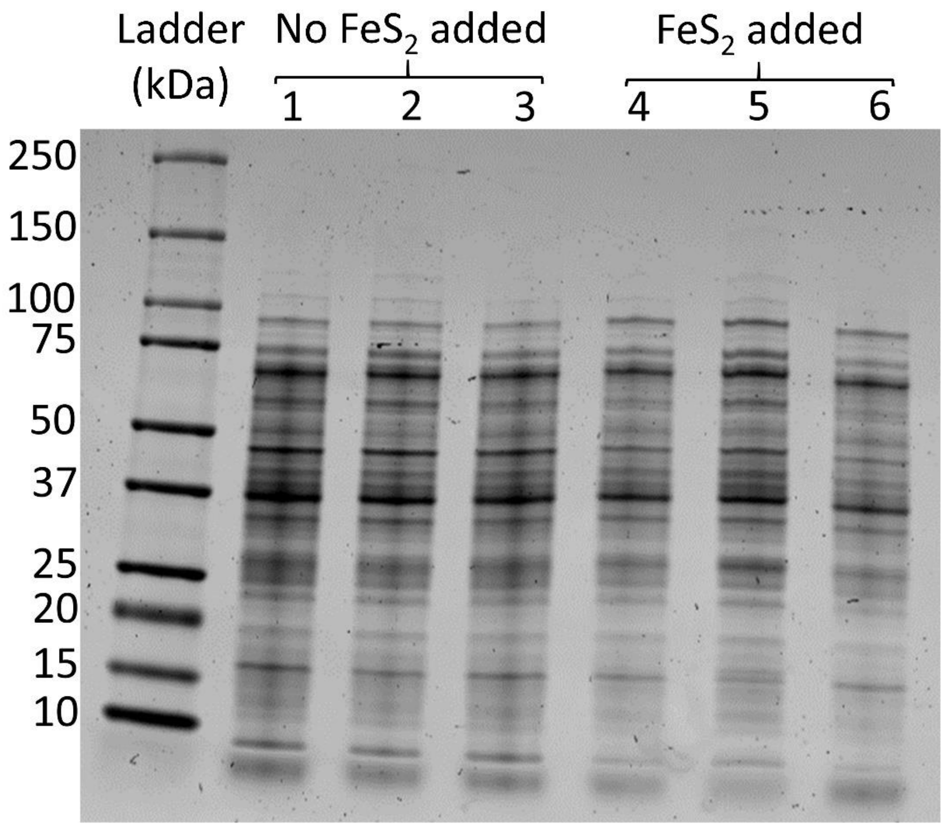

Figure S7. Effect of the presence of pyrite (FeS_2) on the extraction efficiency and recover of protein from *Methanococcus voltae* cells. *M. voltae* was grown with ferrous iron and sulfide in a 2 L reactor with formate as methanogenesis substrate. Mid-log phase cells were concentrated and aliquoted into three tubes with either no mineral added (lanes 1-3) or three tubes with 0.0175 g added pyrite (lanes 4-6). Extracted proteins were subjected to SDS PAGE to qualitatively determine if the presence of FeS_2 influenced the recovery and composition of proteins.

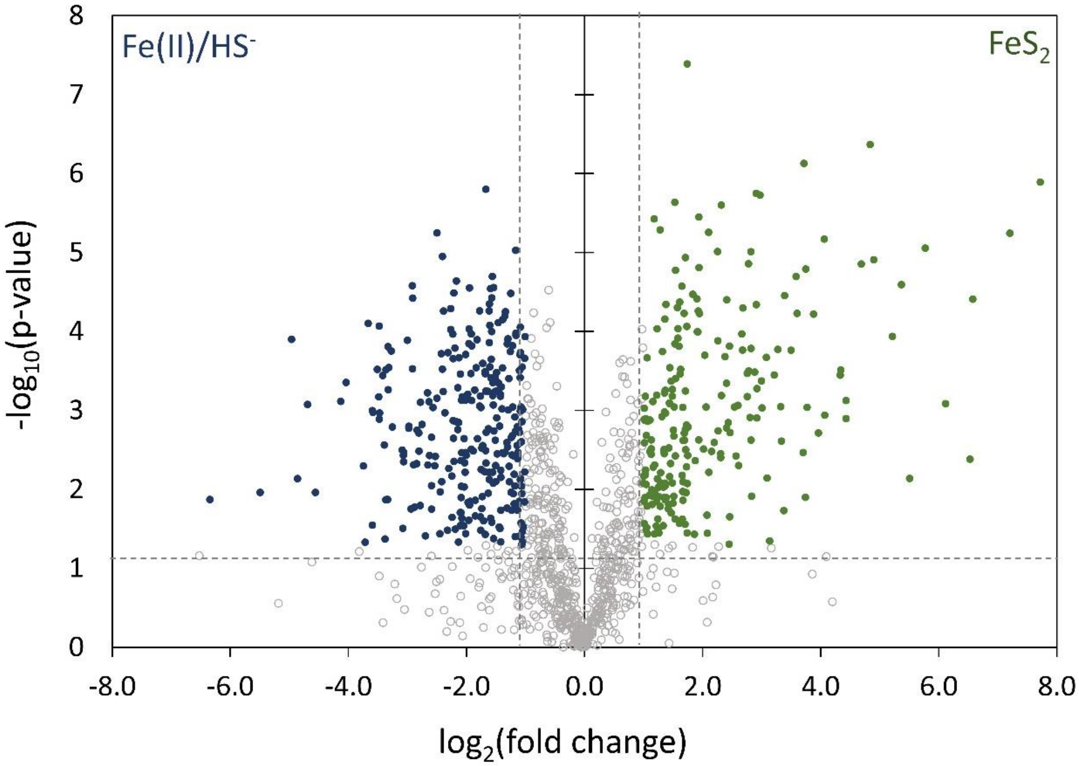

Figure S8. Differential expression of the detected proteome in mid-log phase *Methanococcus voltae* grown with synthetic nanoparticulate pyrite (FeS2) or ferrous iron (Fe(II)) and sulfide (HS^-). The data presented are all proteins identified from the *M. voltae* proteome. Along the x-axis, a positive log_2 fold change (log_2FC) is associated with up-expression of proteins in FeS_2-grown cells, whereas a negative log_2FC is associated with up-expression of proteins in Fe(II)/HS^--grown cells. The significance of differential expression for each protein, calculated as $-log_{10}$(*p*-value), is plotted along the y-axis with increasing values indicating higher statistical significance. Colored bubbles indicate proteins with significant ($p < 0.05$) differential expression with a log_2FC of at least 1. Dashed grey lines show cutoff values for significance and log_2FC.

Table S1. **Cell growth and iron (Fe) data from *Methanococcus voltae* cultures used in atomic absorption (AA) and electron paramagnetic resonance (EPR) spectroscopy experiments.** *M. voltae* was grown with formate and either 26 µM ferrous iron (Fe(II)) and 2 mM sulfide (HS^-) or 2 mM synthetic nanoparticulate pyrite (FeS_2) as the sole Fe and sulfur source. Cells were separated from minerals and concentrated to form a cell pellet that was used for AA and EPR spectroscopic analyses. Fe per cell volume was calculating by dividing total fmol Fe by the total volume of cells under either condition. S=1/2 spin quantitation was determined at 12 K, 10 µW for all spectra in comparison to a Cu(II) standard.

Sample	Culture cell density	Recovered cell density	Total µmoles Fe in AA sample	fmol Fe per cell vol (μm^3)	EPR spin quantitation S=1/2 features (µM Fe)
Fe(II)/HS^- (1)	4.04E+08	1.70E+10	0.121	0.097	17.2
Fe(II)/HS^- (2)	1.72E+08	2.02E+10	0.074	0.087	16.3
Fe(II)/HS^- (3)	2.36E+08	2.07E+10	0.133	0.049	14.5
Fe(II)/HS^- (4)	3.60E+08	5.28E+10	0.197	0.050	16
FeS_2 (1)	1.84E+08	1.49E+10	0.208	0.155	21.6
FeS_2 (2)	2.78E+08	1.55E+10	0.058	0.167	7.8
FeS_2 (3)	3.53E+08	3.78E+10	0.268	0.314	33.2
FeS_2 (4)	4.31E+08	5.64E+10	0.250	0.196	18.7

Table S2. Growth data for *Methanococcus voltae* samples used in proteomics experiments. *M. voltae* was grown with formate and with either 26 µM ferrous iron (Fe(II)) and 2 mM sulfide (HS^-) or 2 mM synthetic nanoparticulate pyrite (FeS_2) as the sole iron and sulfur source. Cultures were harvested in an anaerobic chamber during log phase of growth. Spent supernatant from cultures was used to precipitate soluble proteins.

	**Cells mL^{-1}**	**Methane (ppm)**	**Cellular protein concentration (mg mL^{-1})**
Fe(II)/HS^- (1)	1.64E+08	7.62E+04	27.66
Fe(II)/HS^- (2)	1.21E+08	5.81E+04	14.25
Fe(II)/HS^- (3)	3.07E+08	1.25E+05	29.70
FeS_2 (1)	2.71E+08	1.16E+05	31.56
FeS_2 (2)	1.59E+08	1.03E+05	22.89
FeS_2 (3)	1.71E+08	1.33E+05	28.38

Supplementary Table 3. Components of basal medium (g L^{-1}) and amendments used to cultivate *Methanococcus voltae.*

K_2HPO_4	0.14
KCl	0.33
$MgCl_2 \cdot 6H2O$	5.10
NH_4Cl	0.50
$CaCl_2 \cdot 2H_2O$	0.10
NaCl	21.98
$NaHCO_3$*	5.00
Organics solution**	10.00 mL
Vitamins solution**	10.00 mL
Trace element solution**	10.00 mL

* Added inside an anaerobic chamber
** *See* supplementary materials and methods for recipes
